# Discovery of a Novel Non‐Nucleoside Inhibitor of RNA‐Dependent RNA Polymerase Against Dengue Virus

**DOI:** 10.1002/mco2.70514

**Published:** 2025-11-29

**Authors:** Xue‐Mei He, Li‐Fang Zou, Jing‐Tao Yu, Tang‐Jia Yang, Zi‐Bin Lu, Hui‐Hui Cao, Wen Li, Bing Chen, Wei Zhao, Jian‐Ping Zuo, Lin‐Zhong Yu, Jun‐Shan Liu

**Affiliations:** ^1^ School of Traditional Chinese Medicine Guangdong Provincial Key Laboratory of Chinese Medicine Pharmaceutics Guangdong Basic Research Center of Excellence for Integrated Traditional and Western Medicine for Qingzhi Diseases Southern Medical University Guangzhou China; ^2^ Analytical and Testing Center Jinan University Guangzhou China; ^3^ Guangdong Provincial Key Laboratory of Tropical Disease Research School of Public Health Southern Medical University Guangzhou China; ^4^ Laboratory of Immunopharmacology State Key Laboratory of Drug Research Shanghai Institute of Materia Medica Chinese Academy of Sciences Shanghai China

**Keywords:** AG129 mice, dengue virus, ICR suckling, RNA‐dependent RNA polymerase, target

## Abstract

Dengue virus (DENV) is an acute infectious pathogen worldwide, for which no effective therapeutics are available. The RNA‐dependent RNA polymerase (RdRp) displays an important role during DENV replication and is therefore a promising target in the development of antiviral drugs. However, there are still no clinically approved RdRp inhibitors available. In this study, we identified a natural small molecule, 12β‐hydroxydammar‐3‐one‐20(S)‐O‐β‐d‐glucopyranoside (PN‐1), using a surface plasmon resonance‐based screening assay. Biochemical and structural analyses revealed that PN‐1 selectively targets the RdRp of DENV NS5 protein by covalently modifying residues Glu255, Met387, Glu479, and Ala507. Mechanistic studies involving tryptophan scanning and hydrogen‐deuterium exchange mass spectrometry revealed that PN‐1 binding regulates RdRp conformational transitions. This allosteric mechanism leads to suppression of enzymatic activity and inhibition of DENV replication. Consequently, PN‐1 exhibited potent antiviral activity across various cell lines and conferred significant protection in both ICR suckling and AG129 mouse models. Taken together, our results show that PN‐1 functions as a novel non‐nucleoside inhibitor to suppress DENV replication by targeting RdRp. These findings highlight PN‐1 as a promising anti‐DENV lead compound, while revealing conserved RdRp residues as actionable targets for rational drug design.

## Graphical abstract

PN‐1, a novel non‐nucleoside inhibitor of the dengue virus RNA‐dependent RNA polymerase (RdRp), was identified from Panax notoginseng. This compound selectively targets the DENV‐2 NS5 RdRp, inducing conformational changes that inhibit enzymatic activity, thereby suppressing DENV replication both in vitro and in vivo.

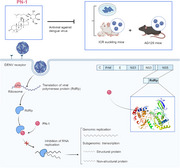

## 1 Introduction

Dengue virus (DENV) is one of the most prevalent arthropod‐borne viral pathogens worldwide and is mainly transmitted by *Aedes aegypti* or *Aedes albopictus* [1]. This virus is divided into four serotypes (DENV1–4) based on differences in surface antigens. Infection with any of these serotypes will produce different clinical symptoms ranging from asymptomatic dengue with and without warning signs to severe dengue [2]. It is estimated that 100–400 million DENV infections occur annually in over 100 countries, resulting in approximately 100 million symptomatic infections, 2 million cases of severe disease, and 20,000 deaths [3, 4, 5]. Since its designation by the World Health Organization (WHO) as one of the 10 threats to global health in 2019, the global incidence of dengue has shown an increasing trend from year to year, with the highest number of cases reported in 2024. The WHO reported a record 14.4 million dengue cases in 2024, leading to over 11,000 fatalities. These cases were predominantly clustered in the Americas, with Brazil recording the highest number, exceeding 10.26 million. Dengue has evolved from a sporadic disease to a public health problem with significant social and economic impact [6]. To date, there are still no clinically approved reagents available, except two approved vaccines CYD‐TDV (Dengvaxia) and TAK‐003. However, higher hospitalization incidence rates and more severe disease in seronegative individuals were observed with CYD‐TDV during long‐term safety studies, which prompted the WHO to restrict the use of the vaccine to individuals seropositive for dengue over the age of 9 [7]. The second approved vaccine TAK‐003, developed by Takeda, exhibited an unbalanced protective response against the four serotypes of DENV [8].

The DENV genome encodes for three structural proteins (capsid C, pre‐membrane/membrane prM/M, and envelope E) and seven non‐structural (NS) proteins (NS1, NS2A, NS2B, NS3, NS4A, NS4B, and NS5) [9]. NS5 is critical for genome replication and RNA capping and is the most conserved among NS proteins, which is comprised of a methyltransferase (MTase) domain at its N‐terminus and an RNA‐dependent RNA polymerase (RdRp) domain at the C‐terminus. The MTase domain mainly accounts for methylating viral genomic RNA, while the RdRp domain is critical for viral RNA synthesis [10, 11, 12]. Following DENV infection, RdRp carries out complementary negative‐stranded RNA synthesis via de novo initiation mechanism with positive‐stranded viral RNA as a template. The former serves as a template to synthesize positive‐stranded viral RNA, which participates in viral protein translation or infectious virion production [13]. In addition, the absence of a homologous counterpart to RdRp in mammalian cells prevents it from causing target‐related side effects. Therefore, the importance of RdRp for viral genomic RNA makes it an attractive target for the development of antiviral against DENV infection [14]. Suppression of RdRp has proven to be a successful antiviral strategy, as evidenced by marketed agents against severe acute respiratory syndrome coronavirus 2 (SARS‐CoV‐2) and hepatitis C virus (HCV) [15, 16, 17]. For example, remdesivir displays antiviral effects against SARS‐CoV‐2 by competing with natural nucleoside triphosphate (NTP) for binding to the viral RdRp, causing the termination of RNA synthesis [15]. Dasabuvir could bind to RdRp active sites and change its conformation to inhibit the enzyme activity, which finally suppresses HCV infection [17].

RdRp inhibitors comprise nucleoside analog inhibitors (NIs) and non‐nucleoside inhibitors (NNIs). NIs compete with natural NTPs or induce RNA chain termination [18], whereas NNIs bind allosteric sites to induce conformational changes of RdRp [19]. Although many DENV RdRp inhibitors are identified, they have been terminated in the preclinical phase due to severe toxicity or lack of significant effects in animals. NITD008, an adenosine analog, was terminated in a preclinical safety study owing to severe side effects, such as weight loss, retching, and movement disorders [20]. Balapiravir, a nucleotide analogue of HCV RdRp, has been repurposed for DENV due to its ability to inhibit the RdRp activity of DENV. However, it failed to demonstrate clinically therapeutic efficacy in a phase II trial, with no significant reductions in viral load or NS1 antigen levels [21].

In this study, we used a chemical genetics strategy to identify a small molecule, 12β‐hydroxydammar‐3‐one‐20(S)‐O‐β‐D‐glucopyranoside (PN‐1), as a novel NNI of RdRp. Further mechanistic studies revealed that PN‐1 induced conformational changes in RdRp, and consequently inhibited the enzyme activity, leading to a decrease of DENV replication in vitro and in vivo. Collectively, our results suggest that PN‐1 could serve as a lead compound for the development of RdRp‐targeted antivirals, also indicate that triterpenoid saponins may be a fertile resource for the discovery of agents for DENV infection therapy.

## 2 Results

### 2.1 Discovery of PN‐1 That Specifically Targets RdRp

**FIGURE 1:**
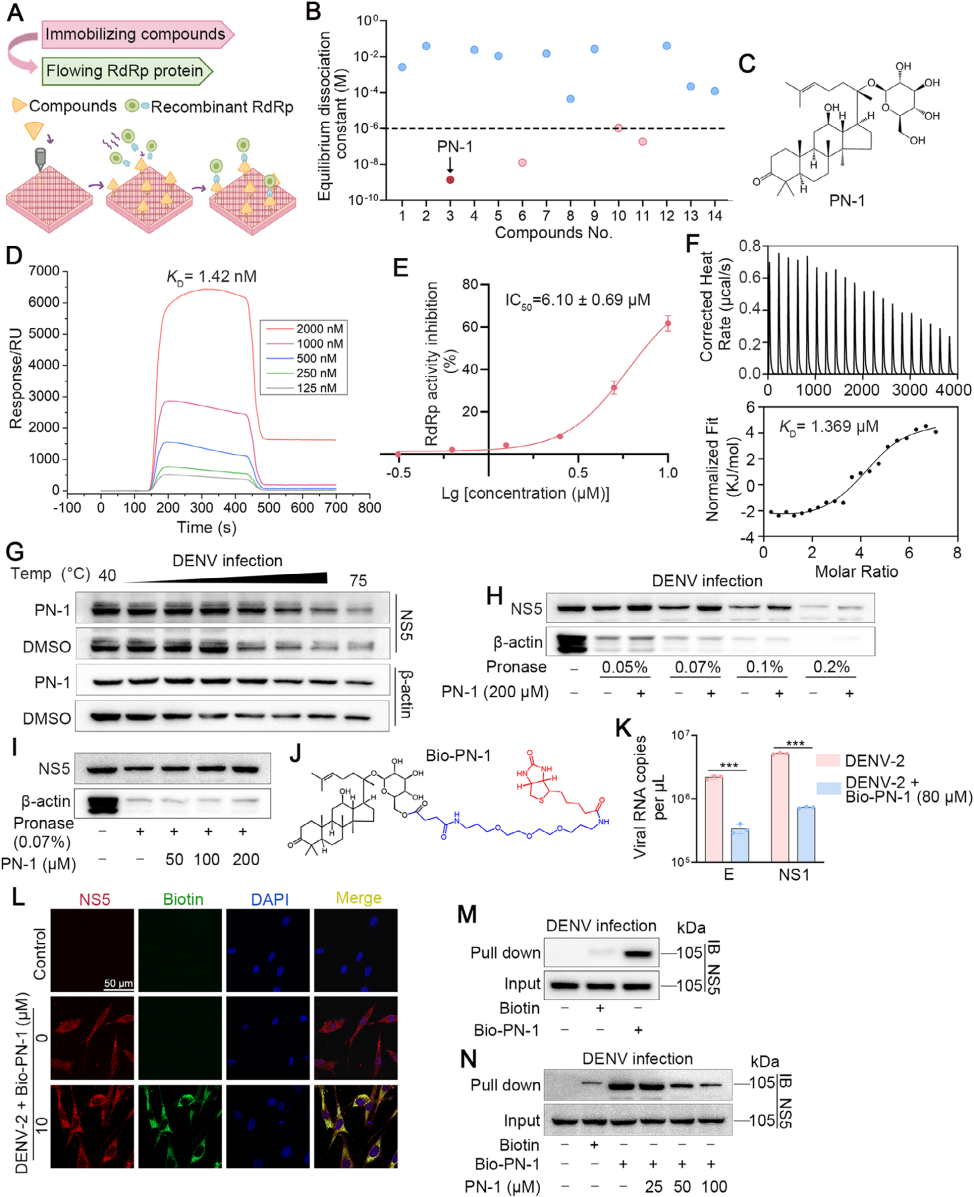
PN‐1 directly targets NS5 RdRp. (A) Schematic representation of SPR screening for compounds interaction with RdRp. (B) *K*_D_ values for the binding of fourteen compounds to RdRp. The black dashed line represents the *K*_D_ below 10^−6^. Red points represent compounds that may interact with RdRp. (C) Chemical structure of PN‐1. (D) The binding affinity of PN‐1 with RdRp determined by SPR assay. (E) The enzymatic activity of RdRp after treatment with PN‐1. (F) The binding kinetics of PN‐1 with RdRp were calculated by ITC analysis. (G–I) Thermal stability and proteolytic susceptibility of NS5 in the presence or absence of PN‐1 determined by CETSA and DARTS assays. (J) Chemical structure of Bio‐PN‐1. (K) Viral RNA copies of E and NS1 with or without Bio‐PN‐1 treatment by qRT‐PCR assay. (L) Co‐localization of Bio‐PN‐1 (Biotin, green) and NS5 (red) detected by immunofluorescence analysis (scale bars = 50 µm). BHK‐21 cells were infected with DENV‐2 for 45 h, then 10 µM Bio‐PN‐1 was added and the culture was continued for additional 3 h (48 h in total), followed by fixation for immunofluorescence experiments. (M, N) Pull down assay was performed using DENV‐2 infected cell lysates. ^***^ *p* < 0.001 versus DENV‐2 model.

Screening antiviral drugs from natural products represents an effective strategy for combating viral infections. Given the established broad‐spectrum antiviral activity of saponins from genus *Panax* against a variety of viruses (e.g., HCV, SARS‐CoV‐2, and influenza) [22, 23, 24, 25, 26, 27], and the taxonomic proximity of HCV and DENV within *Flaviviridae*, we prioritized dammarane‐type triterpenoid saponins from *Panax notoginseng* (Burk.) F. H. Chen as candidates. Specifically, 14 structurally characterized saponins were previously isolated by our group [28], and their chemical structures are shown in Figure S1. Considering the significance of RdRp in the DENV replication cycle, we attempted to identify whether these small molecules could interact with RdRp. For this purpose, we established a screening system using the surface plasmon resonance (SPR) analysis (Figure 1A). We immobilized these compounds on the chip surface and then flowed the RdRp protein to observe their equilibrium dissociation constant (*K*_D_). Four candidates were identified with *K*_D_ less than 10^−6^, implying their potential interaction with RdRp (Figure 1B). Among them, compound **3** (PN‐1) exhibited strong binding affinity toward RdRp, with a *K*_D_ value of 1.42 nM (Figure 1C,D and Figure S2). Considering that RdRp functions as an RNA polymerase and thereby synthesizes viral RNA genomes in a de novo manner, we examined whether the binding of PN‐1 to RdRp affects the enzymatic activity of RdRp. The results showed that PN‐1 effectively inhibited the enzymatic activity of RdRp, with a half maximal inhibitory concentration (IC_50_) of 6.10 µM (Figure 1E). Measurement of PN‐1 interaction with RdRp by isothermal titration calorimetry (ITC) revealed that RdRp bound PN‐1 with *K*_D_ of 1.369 µM (Figure 1F). Next, we performed label‐free target protein identification assays named cellular thermal shift assay (CETSA) and drug affinity‐responsive target stability assay (DARTS). As depicted in Figure 1G and Figure S3A, NS5 protein was degraded gradually with an increase in temperature, while the thermal stabilization of NS5 in the PN‐1‐treated group was increased compared with that in the DMSO group. Moreover, PN‐1 protected NS5 from pronase‐induced degradation in a dose‐dependent manner (Figure 1H,I and Figure S3B,C). Next, we synthesized biotin‐tagged PN‐1 (Bio‐PN‐1) (Figure 1J and Figure S4A,B), which displayed anti‐DENV activity at non‐toxic concentrations (Figure 1K and Figure S5), indicating that it was suitable for labeling. Immunofluorescence analysis revealed that the fluorescence from Bio‐PN‐1 (green) overlapped with that from NS5 (red) in the cytoplasm (Figure 1L). Furthermore, our results showed that NS5 in BHK‐21 cells could be pulled down by Bio‐PN‐1, which was competitively inhibited by increasing concentrations of PN‐1 (Figure 1M,N and Figure S6A,B). The results of molecular docking showed that the docking scores of PN‐1 with NS5 protein were −5.180 kcal/mol (Figure S7).

**FIGURE 2:**
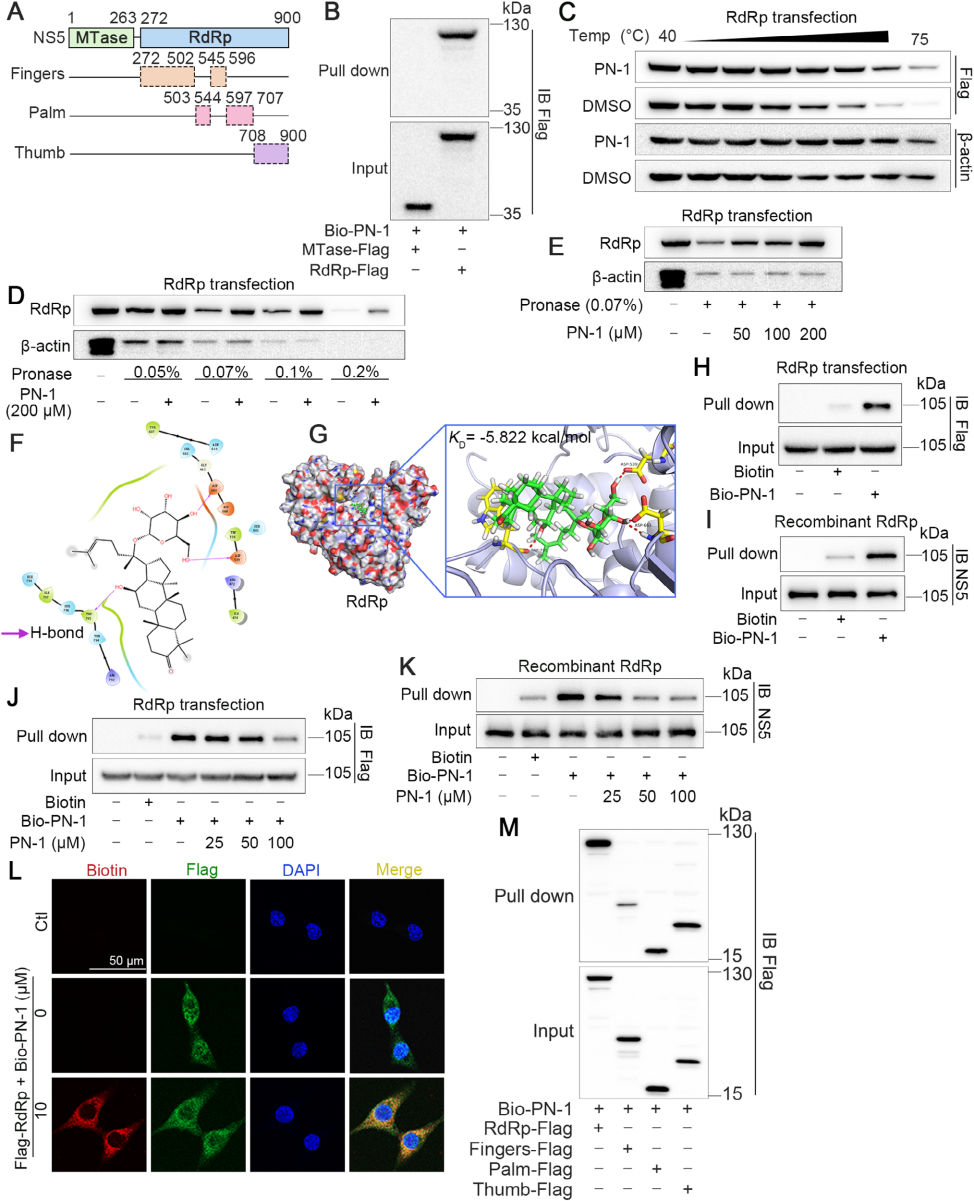
PN‐1 selectively targets RdRp. (A) Schematic of different functional domains of NS5 and subdomains of RdRp. (B) PN‐1 selectively interacted with RdRp domain. (C) Thermal stability of RdRp with or without PN‐1 treatment assessed by CETSA assay. (D, E) Resistance of RdRp to protease by DARTS assay at different concentrations of pronase or PN‐1. (F, G) The binding mode of PN‐1 with DENV‐2 RdRp using molecular docking. (H–K) Pull down assay was performed in RdRp transfected cell lysates or recombinant RdRp with or without PN‐1. (L) Co‐localization of PN‐1 (red) and RdRp (green) detected by immunofluorescence analysis (scale bars = 50 µm). (M) PN‐1 mainly interacted with palm and thumb domains of RdRp.

Since the NS5 protein contains both MTase and RdRp domains (Figure 2A), we further explored whether PN‐1 selectively targets RdRp. We constructed these two plasmids (Figure S8) and performed pull‐down assay. Our results suggested that the NS5 protein could interact with Bio‐PN‐1 despite deletion of the N‐terminal MTase domain, whereas deletion of the C‐terminal RdRp domain counteracted its interaction with Bio‐PN‐1 (Figure 2B). Critically, the results of CETSA and DARTS assays also demonstrated that PN‐1 improved the thermostability and decreased the proteolytic susceptibility of RdRp (Figure 2C–E and Figure S9A–C), while the proteolytic susceptibility of MTase showed no obvious changes after PN‐1 treatment (Figure S10A,B). Molecular docking results confirmed that the *K*_D_ value of PN‐1 bound RdRp was −5.822 kcal/mol, while that of MTase was −5.173 kcal/mol (Figure 2F,G and Figure S10C,D). These results revealed that PN‐1 selectively targets the RdRp domain of NS5.

Next, we transfected the RdRp plasmid into BHK‐21 cells and performed pull down analysis. The results showed that Bio‐PN‐1 captured RdRp from BHK‐21 lysates or recombinant RdRp protein, which phenomenon was competitively inhibited by unlabeled PN‐1 (Figure 2H–K and Figure S11A–D). Fluorescence observation also showed that Bio‐PN‐1 co‐localized with the RdRp plasmid mainly in the cytoplasm (Figure 2L).

RdRp consists of three subdomains: fingers, palm, and thumb (Figure 2A). The subdomains that contributed to the PN‐1 and RdRp interactions were examined by transfection with different truncations of RdRp. Pull down analysis revealed that PN‐1 primarily interacted with the palm and thumb subdomains but not with the fingers subdomain (Figure 2M and Figure S11E). Collectively, these findings suggested that RdRp serves as a direct protein target of PN‐1.

### 2.2 The Amino Acids Glu255, Met387, Glu479, and Ala507 of RdRp Serve as Covalent Binding Sites of PN‐1

**FIGURE 3:**
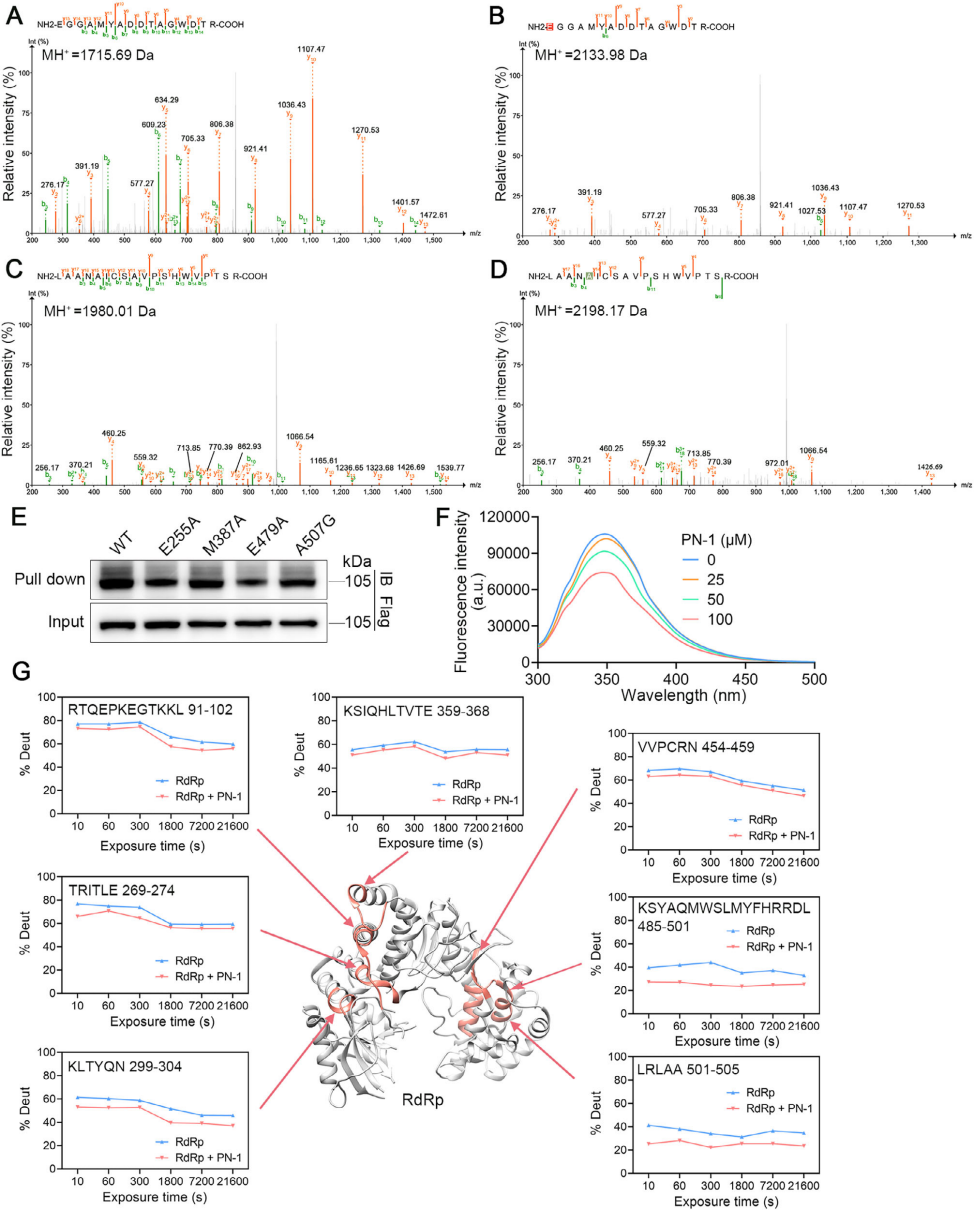
PN‐1 allosterically regulates conformation of RdRp and inhibits its enzyme activity. (A, B) LC‐MS/MS identified PN‐1 covalent binding to site E255 on the peptide EGGAMYADDTAGWDTR. (C, D) LC‐MS/MS revealed the binding site of PN‐1 at the residue A507 on peptide LAANAICSAVPSHWVPTSR. A and C: DMSO control; B and D: PN‐1 treated. Recombinant RdRp protein was incubated with DMSO (left panels) or PN‐1 (right panels) at 4°C for 24 h. Green fragment ion peaks correspond to b‐ions; orange fragment ion peaks represent y‐ions. (E) Pull down analysis was used to detect that E255, M387, E479, and A507 mutation attenuated the interaction between PN‐1 and RdRp. (F) The tryptophan fluorescence intensity of RdRp upon PN‐1 treatment. (G) PN‐1 induced the conformational changes of RdRp determined by HDX‐MS analysis. The peptides with lower levels of hydrogen–deuterium exchange after PN‐1 treatment have been marked in red.

To determine which amino acid residues of RdRp were modified by PN‐1, we conducted LC‐MS/MS analysis by incubating recombinant RdRp protein with PN‐1. The main fragmentation peaks of PN‐1 were F1 and F2, with exact mass of 202.13 and 218.16, respectively. Because the molecular weight of PN‐1 is 620.42, the remaining parts of F1 and F2 are F3 and F4, with precise molecular weights of 418.29 and 402.26, respectively (Figure S12). Therefore, we speculated that the RdRp protein may be covalently modified by these four ions (F1–F4) through specific amino acids. Interestingly, the peptide EGGAMYADDTAGWDTR, with a calculated mass of 1715.69 Da was identified. The mass of the peptide increased by 418.29 Da (2133.98 Da) after incubation with PN‐1, which accurately matched the molecular weight of the fragmentation peak F3. MS/MS analysis of this peptide showed that a 418.29 Da mass shift (from b1 fragment ions), suggesting that the glutamic acid in this peptide (E255 in RdRp) was modified by PN‐1 (Figure 3A,B). Moreover, the measured mass of LAANAICSAVPSHWVPTSR peptide was 1980.01 Da in the absence and 2198.17 Da in the presence of PN‐1, the mass difference between them (218.16 Da) was consistent with the molecular weight of the main fragmentation peak F2. MS/MS spectra revealed that a 218.16 Da mass shift from y15 to y18 fragment ions, demonstrating that alanine in this peptide (A507 in RdRp) was involved in PN‐1 interaction with RdRp (Figure 3C,D). Similarly, we identified two other peptides MAISGDDCVVKPLDDR and ETACLGK, which were specifically modified by PN‐1 on Methionine387 (M387) and E479, respectively (Figure S13A–D).

To confirm that these four residues are the binding sites of PN‐1 for RdRp, we mutated E255 into A255 (E255A), M387 into A387 (M387A), E479 into A479 (E479A), and A507 into G507 (A507G). Our data showed that these mutant plasmids were successfully overexpressed in BHK‐21 cells (Figure S14), and that the amount of mutant RdRp protein pulled down by PN‐1 was significantly decreased (Figure 3E and Figure S15). These observations demonstrated that the above residues serve as crucial covalent binding sites for PN‐1.

### 2.3 PN‐1 Induces Conformational Changes of RdRp

To study the effects of PN‐1 on RdRp conformation, we first performed tryptophan fluorescence analysis using recombinant RdRp protein. The fluorescence intensity of tryptophan in RdRp decreased upon PN‐1 treatment, indicating the conformational change of RdRp (Figure 3F). Next, we explored the dynamic changes in RdRp using hydrogen‐deuterium exchange‐mass spectrometry (HDX‐MS). We achieved an RdRp peptide coverage of > 90% (Figure S16). Hydrogen‐deuterium exchange in the presence of PN‐1 decreased in the fingers subdomain (peptides 91‐102 and 299‐304), palm subdomain (peptides 269‐274 and 359‐368), and thumb subdomain (peptides 454‐459, 485‐501, and 501‐505) (Figure 3G and Figure S17A). The most significant HDX reductions localize to two adjacent segments within the thumb domain (peptides 485‐501 and 501‐505), indicating that PN‐1 primarily alters the conformation of this domain and thereby inhibits RdRp activity.

Moreover, increased hydrogen‐deuterium exchange was observed in fingers (peptides 57‐71, 74‐89, 103‐108, 125‐140, and 143‐152) and palm subdomains (peptides 234‐248) (Figure S17B). The increase in hydrogen‐deuterium exchange may be interpreted as a consequence of obvious conformational changes of RdRp induced by PN‐1, resulting in the exposure of these regions. Taken together, these results suggested that PN‐1 induces conformational changes and further decreases the enzymatic activity of RdRp.

### 2.4 PN‐1 Inhibits DENV Infection In Vitro

**FIGURE 4:**
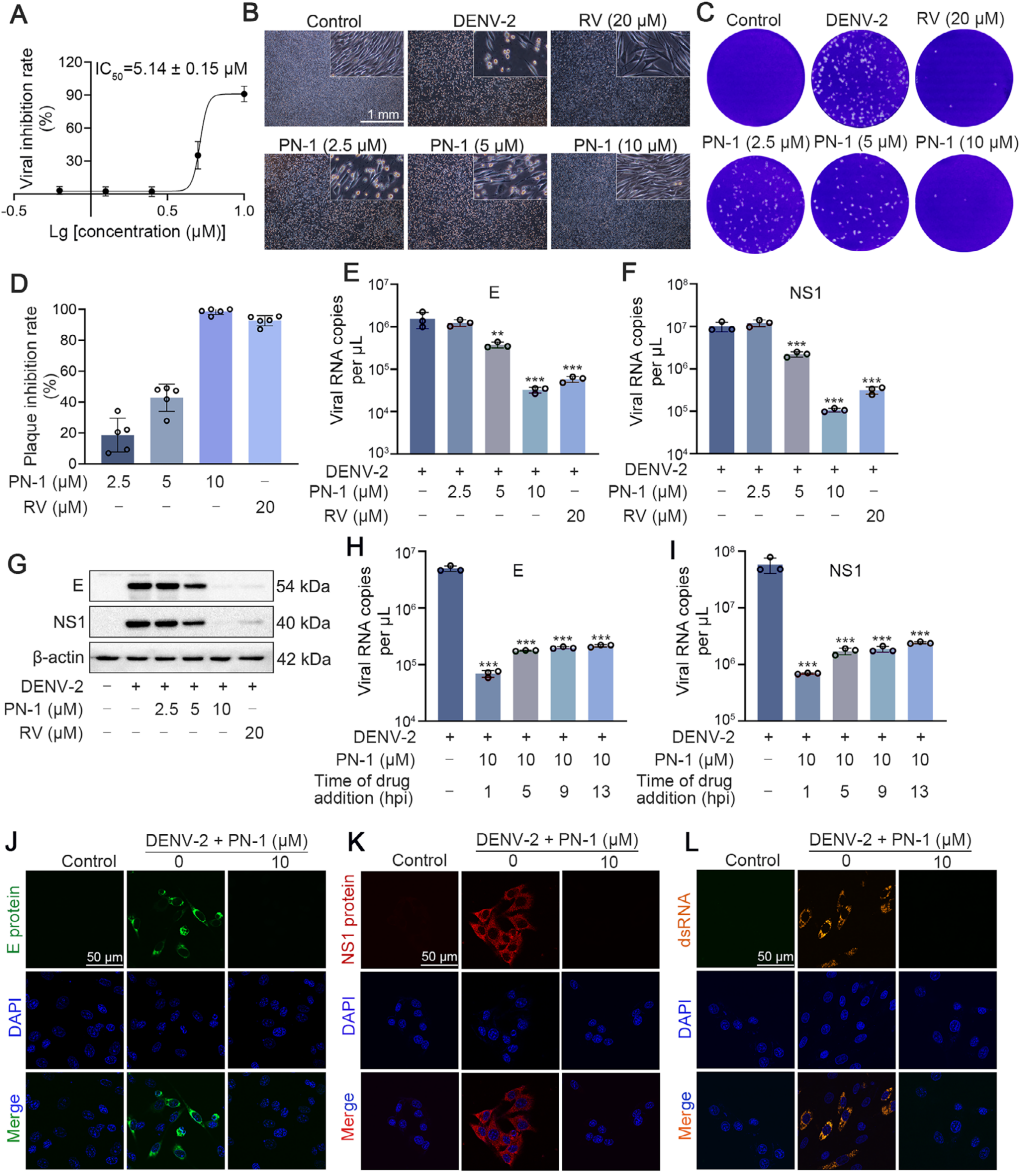
PN‐1 inhibits DENV replication. (A) Inhibition rate of PN‐1 on BHK‐21 cells against DENV‐2 intracellular replication. After DENV‐2 infection and PN‐1 treatment, BHK‐21 cell viability was quantified using the CCK‐8 assay, and viral inhibition rate was calculated. (B) PN‐1 improved the CPE of BHK‐21 cells induced by DENV‐2. The cells were photographed under an IX 53 light microscope. (C, D) Viral progeny synthesis in DENV‐2‐infected BHK‐21 cells with or without PN‐1. (E‐G) Quantification of viral E and NS1 RNA copies and protein levels in DENV‐2‐infected BHK‐21 cells by qRT‐PCR and western blot analysis. (H, I) Quantification of viral E and NS1 RNA copies when PN‐1 was added at different points. PN‐1 was administered at specific timepoints post‐infection (1, 5, 9, and 13 hpi), and cellular RNA was harvested at 48 hpi for qRT‐PCR analysis. (J–L) The expression of E (green), NS1 (red), and dsRNA (orange) detected by immunofluorescence assay (scale bars = 50 µm). ^**^ *p* < 0.01, ^***^ *p* < 0.001 versus DENV‐2 model.

We next evaluated the antiviral effect of PN‐1. The cytotoxicity of PN‐1 on BHK‐21 cells was determined using MTT assay and non‐toxic doses of 10 µM and below were selected (Figure S18A). We first explored the stage that was affected by PN‐1 during the DENV life cycle. PN‐1 had no obvious effect on DENV‐induced cell death when administered pre‐infection or at viral adsorption or entry phases (Figure S18B–D). In contrast, viral infection was significantly inhibited when PN‐1 was added 1 h post‐infection (hpi), with an IC_50_ value of 5.14 µM (Figure 4A). These results suggest that PN‐1 may inhibit DENV replication but exerts little effect on DENV attachment and entry.

Therefore, we further evaluated the effects of PN‐1 on DENV‐2 replication using ribavirin (RV) as the positive agent [29]. After DENV infection, BHK‐21 cells showed obvious cytopathic effects (CPE), including cell detachment, cell shrinkage, and ultimately death, whereas PN‐1 significantly decreased DENV‐2‐induced CPE, indicating the protective effects of PN‐1 on BHK‐21 cells (Figure 4B). Plaque assay data showed that PN‐1 remarkably decreased plaque formation induced by DENV‐2 in BHK‐21 cells, suggesting that viral progeny synthesis was inhibited (Figure 4C,D). Next, the viral RNA and protein expression levels of E and NS1 were analyzed using quantitative real‐time PCR (qRT‐PCR) and western blot assays. Similar to the effect of RV, PN‐1 significantly inhibited RNA expression and protein levels of E and NS1 (Figure 4E–G and Figure S19A,B). After entering cells, DENV genomic RNA is translated into protein, RNA is synthesized, and progeny virions are assembled and released. To explore the specific stage affected by PN‐1, we performed a time‐of‐drug‐addition assay. The results showed that treatment with PN‐1 at 1 hpi significantly reduced viral RNA copies, whereas administration at 5, 9, and 13 hpi resulted in progressively diminished antiviral efficacy (Figure 4H,I). Immunofluorescence confirmed strong cytoplasmic signals of E and NS1 proteins in DENV‐2‐infected BHK‐21 cells, whereas PN‐1 treatment reduced these signals to nearly undetectable levels (Figure 4J,K). Double‐stranded RNA (dsRNA), which is synthesized during replication and transcription [30], also markedly decreased after PN‐1 treatment (Figure 4L). These results suggest that PN‐1 exerts antiviral effects against DENV‐2 at an early stage of replication.

**FIGURE 5:**
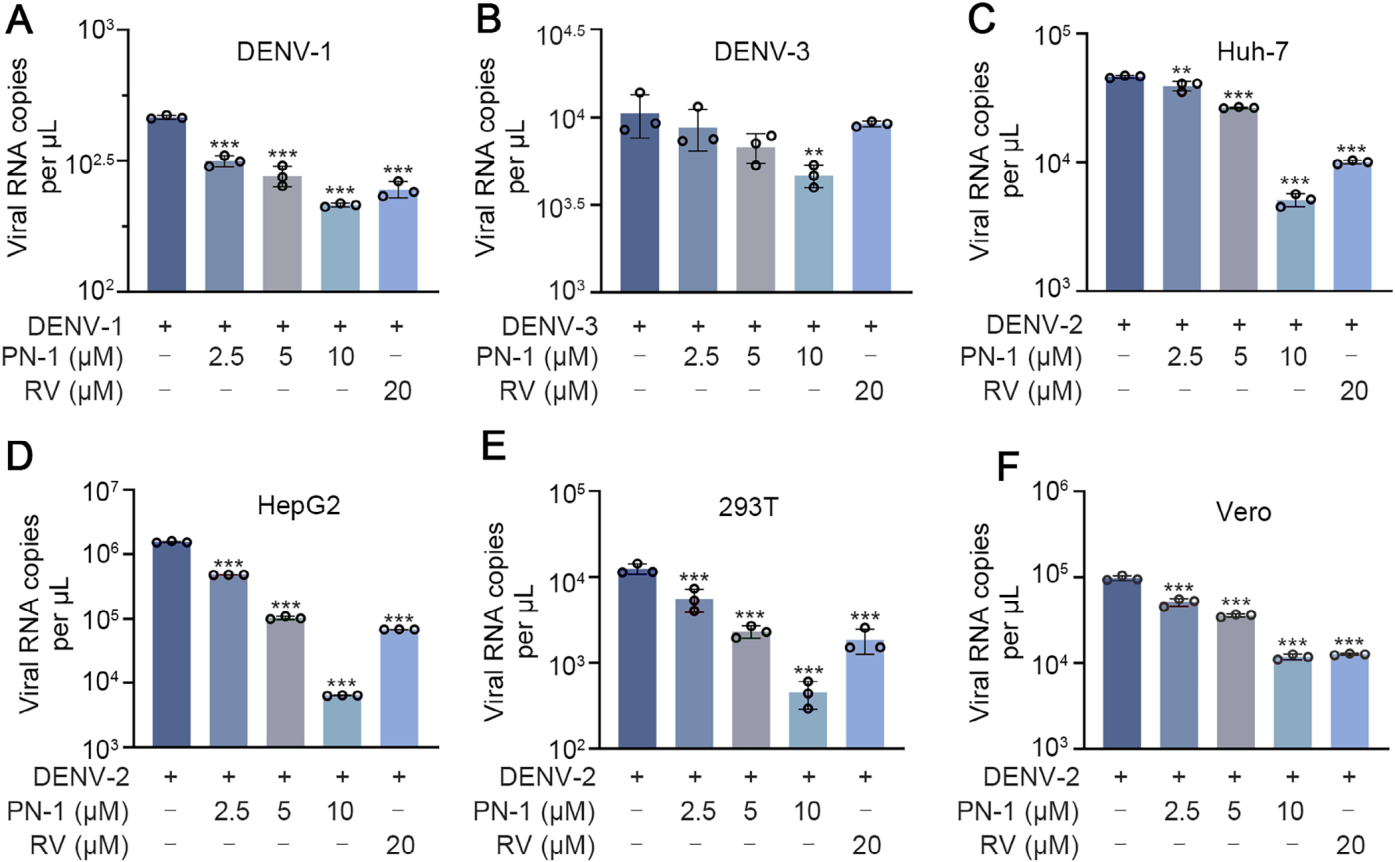
PN‐1 exerts anti‐DENV activities in various DENV serotypes and cell lines. (A, B) Quantification of DENV‐1 NS1 and DENV‐3 NS5 RNA copies after PN‐1 treatment in BHK‐21 cells. (C–F) Effects of PN‐1 on DENV‐2 E RNA copies in Huh7, HepG2, 293T, and Vero cells. ^**^ *p* < 0.01, ^***^ *p* < 0.001 versus DENV model.

Moreover, the pan‐serotype activities of PN‐1 were evaluated by infection of DENV‐1 strain Hawaii and DENV‐3 strain H87 in BHK‐21 cells. PN‐1 showed inhibitory effects on the viral RNA copies of DENV‐1 and DENV‐3 (Figure 5A,B). Since DENV is transmitted to humans through mosquito bites, we further evaluated the antiviral activities of PN‐1 in different cell lines, including human‐derived Huh7, HepG2, 293T cells, and monkey‐derived Vero cells. MTT results showed that PN‐1 at concentrations of 10 µM and below did not affect the proliferation of Huh7, HepG2, 293T, and Vero cells (Figure S20). Similar to the results in BHK‐21 cells, PN‐1 significantly suppressed viral RNA expression in these susceptible cells, especially at a concentration of 10 µM, which was better than or the same as that of RV (Figure 5C–F). Collectively, these results demonstrated that PN‐1 significantly inhibits DENV infection in vitro.

### 2.5 PN‐1 Inhibits DENV‐2 Infection in ICR Suckling and AG129 Mice

**FIGURE 6:**
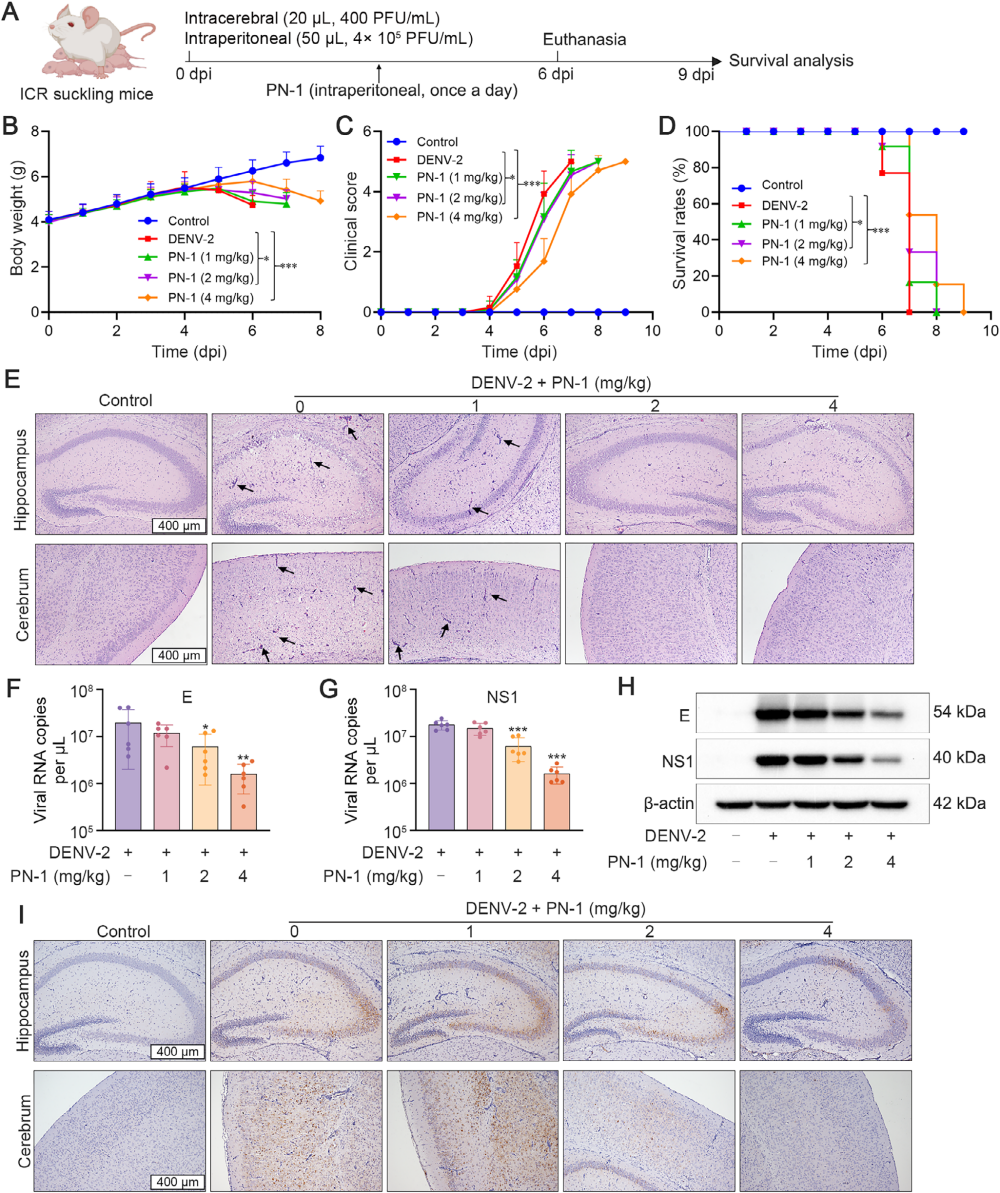
Protective effects of PN‐1 against DENV‐2 infection in ICR suckling mice. (A) The scheme of DENV‐infected ICR suckling mice experiment: mice were intracranially and intraperitoneally inoculated with DENV‐2 and further intraperitoneally administered with PN‐1 for 6 consecutive days. (B) Daily body weight of infected or mock‐infected mice. (C) Clinical scores of infected or mock‐infected mice. (D) Survival rates after infection with DENV‐2 and treatment with PN‐1. (E) Representative images of H&E staining in brain cortex and hippocampus (scale bars = 400 µm). The black arrow represents the perivascular cell infiltration. (F, G) Quantification of viral E and NS1 RNA copies in DENV‐2 infected ICR mice brain. (H) Viral protein levels of E and NS1 in ICR mice brain determined by western blot analysis. (I) Representative images of IHC staining for E in brain cortex and hippocampus (scale bars = 400 µm). ^*^ *p* < 0.05, ^**^ *p* < 0.01, ^***^ *p* < 0.001 versus DENV model.

PN‐1 at non‐toxic dosages was used to evaluate its protective effects against DENV infection in ICR suckling mice (Figure 6A and Figure S21). After DENV infection, the mice exhibited obvious pathological changes, including ruffled fur, body weight loss, limb weakness, paralysis, and eventually death. In contrast, PN‐1 treatment significantly attenuated weight loss, decreased clinical scores, and prolonged survival times (Figure 6B–D). Hematoxylin and eosin (H&E) staining revealed that neurons in the control group maintained normal cytoarchitecture, characterized by tightly aligned arrangement, and clearly defined hippocampal boundaries. In contrast, DENV infection induced severe histopathological alterations, including disrupted neuronal organization, marked decrease of neurons in the cortex and hippocampus, and pronounced perivascular cell infiltration (black arrows). Importantly, PN‐1 treatment attenuated these pathological changes (Figure 6E). Moreover, the RNA and protein expression levels of E and NS1 in the brain decreased after PN‐1 treatment (Figure 6F‐I and Figure S22A,B). However, no significant pathological changes were observed in other tissues, including the heart, liver, spleen, lung, and kidney (Figure S23).

**FIGURE 7:**
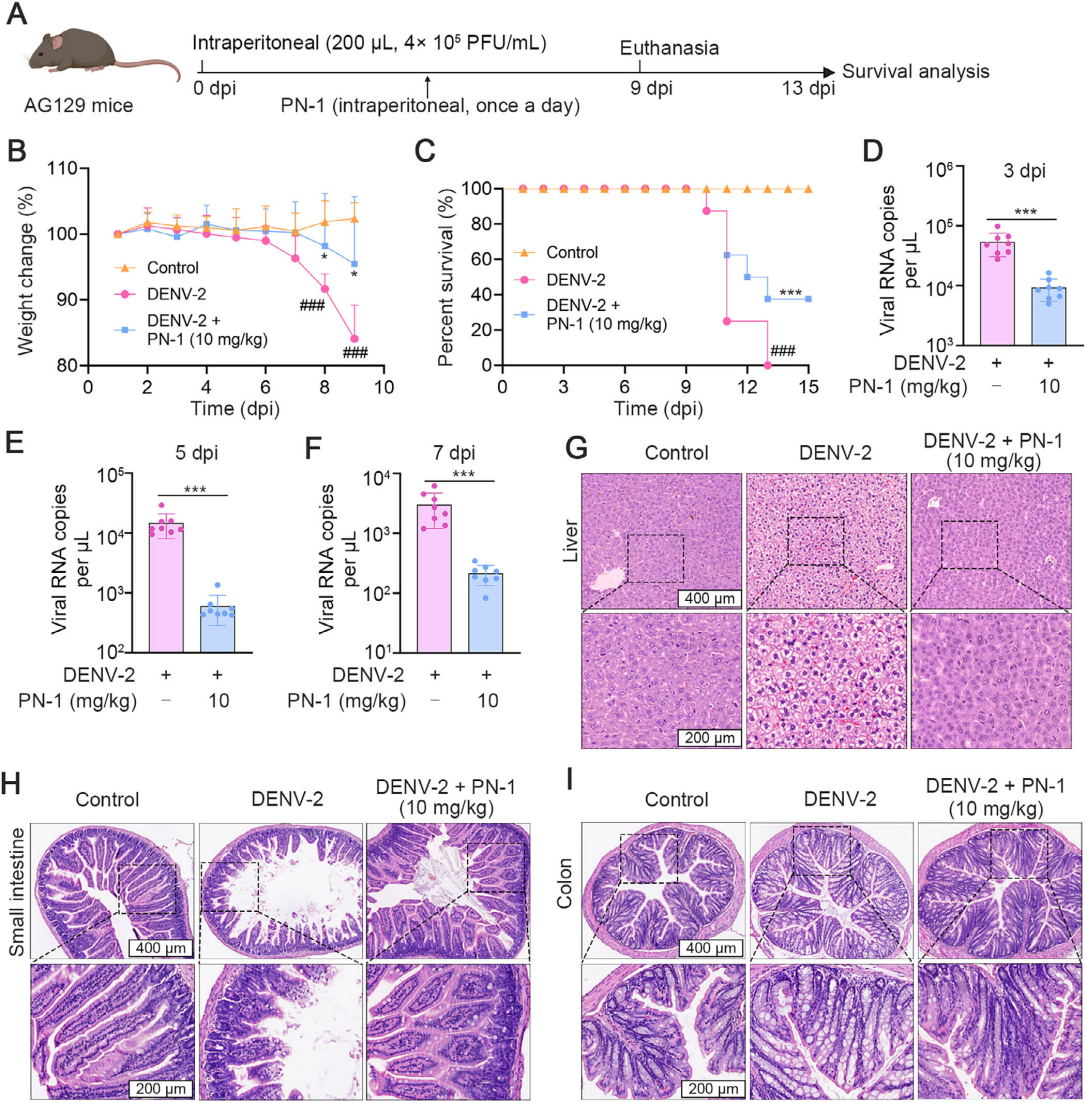
Protective effects of PN‐1 against DENV‐2 infection in AG129 mice. (A) The scheme of DENV‐infected AG129 mice experiment. (B) Daily body weight of infected or mock‐infected AG129 mice. (C) Survival rates of AG129 mice after infecting with DENV‐2 and treating with PN‐1. (D–F) Viral RNA copies detected by qRT‐PCR assay on days 3, 5, and 7 post‐inoculation. (G–I) Representative H&E staining images of liver, small intestine, and colon tissues (scale bars = 400/200 µm). ^###^ *p* < 0.001 versus control group, ^*^ *p* < 0.05, ^***^ *p* < 0.001 versus DENV model.

The 129/Sv mice deficient in IFN‐α/β and IFN‐γ receptors (AG129 mice) have been extensively used for preclinical testing of antiviral drugs and vaccines, since immunocompromise would lead to a murine environment that supports DENV replication and translation [31, 32]. To further evaluate the efficacy of PN‐1 in adult mice, a DENV infection model in AG129 mice was established by intraperitoneal injection of DENV (Figure 7A). The weight loss was observed in the AG129 mice after DENV infection, which was attenuated by PN‐1 (Figure 7B). Moreover, PN‐1 significantly improved the survival of infected mice (Figure 7C). Previous reports have revealed that AG129 mice infected with DENV displayed systemic infection characterized by high levels of viremia, central nervous system, gastrointestinal tract, and liver damage [33]. Thus, serum was collected at days 3, 5, and 7 after infection to assess the viral load by qRT‐PCR. As shown in Figure 7D–F, PN‐1 treatment significantly decreased the viral RNA copy numbers at these time points, indicating that PN‐1 alleviated DENV‐induced viremia. Moreover, histological analysis revealed that hepatocytes in the control group maintained structural integrity, characterized by uniform cellular dimensions, well‐defined nuclear boundaries, and regular nuclear contours. In contrast, DENV‐2 infection induced significant pathological alterations, including hepatocyte swelling, increased cellular volume, and focal cytoplasmic vacuolation, which was effectively reversed by PN‐1 treatment (Figure 7G). In addition, PN‐1 alleviated villus tip lesions and structural disruption in the small intestine, and goblet cell hyperplasia and hyperproliferation in the colon (Figure 7H,I). Collectively, these results indicated that PN‐1 effectively inhibits DENV‐mediated infection in vivo.

## 3 Discussion

Dengue is a major global health problem with an urgent need for effective treatment and preventive measures. The diversity of serotypes and antibody‐dependent enhancement of DENV has led to the slow process in developing effective anti‐DENV drugs. Although several repurposed agents with antiviral efficacies (chloroquine, balapiravir, celgosivir, and lovastatin) have entered clinical trials, none of them have been shown to display obvious effects in reducing plasma viremia or preventing complications [34]. Recently, emerging dengue therapeutics with distinct mechanisms show clinical promise. JNJ‐1802, a pan‐serotype oral NS3/NS4B inhibitor, demonstrated antiviral activity against DENV‐3 in Phase 2a challenge trials, alongside favorable safety in Phase 1/2a studies [35]. ISLA‐101, targeting NS5‐IMPα/β1‐mediated nuclear trafficking, reduced dengue‐related mortality by 70% and viral loads in its Phase 2a/b PROTECT trial [36]. While these agents show clinical potential, further validation of their antiviral efficacy remains essential, underscoring the continued importance of developing effective dengue therapeutics.

RdRp is the most important target due to its essential role in DENV genome replication, offering reduced off‐target risks compared to host‐directed antivirals [37, 38]. NIs as one kind of RdRp inhibitors, require conversion to active triphosphate forms by host kinases, but their efficacy is challenged by variable kinase activity and competition with intracellular NTP pools [39, 40]. This limitation drives the pursuit of NNIs as promising DENV therapeutics. Of note, previous research identified NITD‐29 that binds to the allosteric site N‐pocket of DENV RdRp, hindering the conformational change of RdRp from initiation to elongation, thus inhibiting the replication of four DENV serotypes in infected cells [12]. In this work, through tryptophan scanning and HDX‐MS experiments, we demonstrated that PN‐1, as a novel NNI, modulates the conformational dynamics of the RdRp thereby inhibiting its enzymatic activity. These data indicate that the binding of PN‐1 to NS5 and its subsequent inhibition of RdRp activity are mediated through an allosteric mechanism.

Although NNIs are likely to have relatively low cytotoxicity, the amino acid residues that form allosteric sites in RdRp are generally susceptible to mutations, leading to the rapid development of drug resistance. However, we found that PN‐1 interacts with peptides 91‐102 (RTQEPKEGTKKL), 269‐274 (TRITLE), and 454‐459 (VVPCRN) in RdRp, all of which contain strictly conserved residues by HDX‐MS analysis. These results suggest that PN‐1 provides a higher barrier advantage for the development of resistance compared to other NNIs. Moreover, the residues 91‐102 in RdRp interacted with PN‐1 are located in functional nuclear localization signals (NLSs). Based on our imaging data showing restricted nuclear accumulation of NS5, and considering that targeting NLSs may attenuate viral replication [41, 42], we propose a second mechanism: beyond direct allosteric inhibition, PN‐1 may also prevent NS5‐mediated dampening of the host response, thereby contributing to its overall antiviral effect. In summary, our study provides compelling evidence that inhibition of RdRp enzymatic activity plays an important role in the antiviral activity of PN‐1 against DENV. However, the potential contribution of interference with NS5 nuclear accumulation to PN‐1's antiviral efficacy remains to be further investigated. In addition, RdRp is relatively conserved across all RNA viruses, indicating that PN‐1 has antiviral potential against other RNA viruses, such as zika virus, HCV, and chikungunya virus.

Given that PN‐1 acts as an NNI of RdRp, we next tested its antiviral activity against DENV in vitro and in vivo. Our results showed that PN‐1 inhibited DENV replication in different DENV serotypes and cell lines, indicating the broad anti‐DENV activities of PN‐1. Moreover, the DENV‐infected ICR suckling mouse model, which has often been adopted to evaluate the protective effects of drugs and vaccines [43], was initially used to explore the antiviral effects of PN‐1 in vivo. Our results suggested that PN‐1 could protect against DENV‐induced loss of neurons in the cortex and hippocampus, whereas no significant pathological changes were observed in other organs. This may be due to the high viral load in the brain, and the mice died before other organs showed pathological changes. Because the route of viral inoculation is less relevant to natural human infection, and pathological changes in ICR suckling mouse brains are not the predominant manifestation of human infection, we subsequently used AG129 mice, which lack both IFNα/β and IFNγ receptors, to further determine the protective effects of PN‐1. Our results showed that AG129 mice had obvious systemic infection symptoms after inoculation, including high levels of viremia and severe organ damage, which could be attenuated after PN‐1 treatment. Previous studies have reported that NIs are prone to produce severe toxicity. However, PN‐1 not only showed remarkable anti‐DENV effects in these two animal models, but also exhibited lower toxicity as reflected by body weight and histopathological examination, implying that PN‐1 may circumvent the drawbacks associated with NIs and hold great potential against DENV infection.

Besides DENV‐2, other serotypes are also important for DENV transmission. This study mainly focused on the interaction of PN‐1 with RdRp of DENV‐2; further investigation is still required to determine the interaction of PN‐1 with RdRp of other DENV serotypes. In addition to the potential viral target RdRp studied here, it is also necessary to test whether PN‐1 interacts with host cell proteins crucial for viral replication in future studies.

In summary, we first identified a small molecule PN‐1 that directly targets RdRp. Critically, PN‐1 induces the conformational changes and further decreases the enzyme activity of RdRp, therefore significantly inhibiting DENV infection in vitro and in vivo. These results indicate PN‐1 may serve as a potential agent for DENV infection treatment, and also provide new insights into the design of lead compounds targeting these conserved residues of RdRp for DENV infection therapy.

## 4 Materials and Methods

Detailed descriptions of the materials (chemicals, viruses) and methods (cell culture, molecular docking, MTT assay, qRT‐PCR, western blot analysis, and immunofluorescence assay) are provided in the Supporting Information.

### 4.1 Recombinant RdRp Protein Expression and Purification

Recombinant DENV‐2 RdRp with a 6 × His‐tag fused to its C‐terminus was cloned into BamHI/XhoI sites of the pET28a vector. *Escherichia coli* strain BL21 (DE3) was transformed with the plasmid and cultured in LB medium at 37°C and 160 rpm overnight. Subsequently, cells were induced with 0.5 mM isopropyl β‐D‐1‐thiogalactopyranoside (IPTG) at 20°C for 12 h. Collected cells were then lysed with lysis buffer (50 mM phosphate‐buffered saline [PBS] [pH 7.4] and 0.15 M NaCl) and disrupted by sonication. The cell pellets were harvested by centrifugation and dissolved in urea. After loading onto a pre‐equilibrated NTA purified column, recombinant RdRp was eluted with NTAU‐0 buffer (NTAU‐X: 50 mM PBS [pH 7.4], 0.15 M NaCl, 8 M Urea, X mM imidazole) and then sequentially eluted with NTAU‐20, NTAU‐50, NTAU‐100, NTAU‐200, and NTAU‐500 buffers. Finally, the protein was concentrated and visualized by sodium dodecyl sulfate‐polyacrylamide gel electrophoresis (SDS‐PAGE) followed by Coomassie blue staining (Solarbio, Beijing, China).

### 4.2 SPR for Screening Compounds That Bind to RdRp

SPR screening was conducted using the PlexArray HT A100 system (Plexera Bioscience, Woodinville, WA, USA), as previously reported [44]. Briefly, 14 dammarane‐type triterpenoid saponins (10 mM) were immobilized on the surface of a 3D photo‐cross‐linking chip, followed by vacuum drying for 30 min. The chips were subjected to photo‐cross‐linking reaction (365 nm) for 15 min and then washed with N,N‐dimethylformamide, anhydrous ethanol, and H_2_O. Different concentrations of recombinant RdRp protein diluted with running buffer PBS were superfused at a rate of 2 µL/s for 300 s to allow for association, followed by 300 s for dissociation. The binding curves and affinity of drug–protein interactions were evaluated using BIAevaluation software (version 4.1). The background interference (nonspecific adsorption and instrument noise) was eliminated to ensure accurate *K*_D_ determination.

### 4.3 DENV‐2 RdRp Assay

The activity of DENV RdRp enzyme was tested using RNA polymerase assay kit following the manufacturer's instructions (ProFoldin, Hudson, MA, USA). The experimental groups were established as follows: (1) The “0% inhibition” control (RdRp + substrate + DMSO, the vehicle); (2) the “100 % inhibition” control (substrate + DMSO, omitting RdRp); (3) different concentrations of PN‐1 treatment (RdRp + substrate + PN‐1 at 0.3125, 0.625, 1.25, 2.5, 5 and 10 µM). A premix composed of the buffer, template, and purified RNA polymerase was incubated with PN‐1 for 10 min, then the substrate NTPs were added to initiate the enzyme reaction. Next, the fluorescence dye was added and incubated for 30 min. The fluorescence intensity was measured at 535 nm using the excitation wavelength at 485 nm. Raw fluorescence values were normalized by subtracting the “100 % inhibition” control signals.

### 4.4 ITC Analysis

ITC experiments were conducted using a NANO ITC instrument (Waters, Milford, MA, USA) at 25°C. RdRp (10 µM) in a calorimetric cell was titrated with PN‐1 (100 µM) 20 times with an equilibrium interval of 200 s. The *K*_D_, enthalpy (Δ*H*), entropy (Δ*S*), and stoichiometry of binding (*n*) were determined using a nanoAnalyzer (Waters).

### 4.5 Cellular Thermal Shift Assay

Cellular Thermal Shift Assay (CETSA) was performed using a standard protocol [45]. DENV‐2‐infected or RdRp plasmid‐transfected BHK‐21 cells were lysed with RIPA lysis buffer and freeze‐thawed twice using liquid nitrogen. After centrifugation for 20 min at 20,000 × *g*, cell lysates were collected and incubated with PN‐1 or DMSO for 1 h at room temperature (RT). Subsequently, the samples were aliquoted into PCR tubes and heated at different temperatures via an Applied Biosystems PCR instrument (Thermo Fisher Scientific, Waltham, MA, USA). The supernatants were collected after centrifugation and analyzed by western blotting.

### 4.6 Drug Affinity‐Responsive Target Stability Assay

The Drug Affinity‐Responsive Target Stability Assay (DARTS) assay was performed as previously described [46]. Briefly, BHK‐21 cells were lysed with M‐PER lysis buffer (Thermo Fisher Scientific), and cell lysate concentrations were quantified using a BCA assay kit (Thermo Fisher Scientific). The lysates were diluted with 1×TNC buffer (500 mM Tris‐HCl [pH 8.0], 500 mM NaCl, and 100 mM CaCl_2_), followed by incubation with PN‐1 or DMSO for 1 h at RT. Subsequently, pronase diluted with 1× TNC solution was added and incubated for 15 min at RT. The loading buffer was then added to the samples to stop digestion. Finally, the samples were analyzed by western blotting.

### 4.7 Pull Down Analysis

To further confirm the interaction between RdRp and PN‐1, we synthesized a biotin‐labeled PN‐1 probe (Bio‐PN‐1). BHK‐21 cell lysates or recombinant RdRp protein were incubated with biotin or Bio‐PN‐1 in the presence or absence of excess PN‐1 overnight at 4°C. After washing with TBST six times, streptavidin magnetic beads were added to the lysates. Subsequently, the samples were incubated overnight at 4°C and placed in DynaMag magnets (Invitrogen, Grand Island, NE, USA) to separate magnetic beads from cell lysates. After washing with TBST six times, IgG elution buffer (pH 2.0, Thermo Fisher Scientific) was added and mixed with the magnetic beads at RT for 5 min. Finally, the samples were placed in DynaMag magnets to magnetically separate the beads, and the supernatants containing the target protein were collected for western blotting.

### 4.8 Plasmid Transfection Analysis

Plasmids overexpressing wild‐type MTase, RdRp, and mutated RdRp (E255A, M387A, E479A, and A507G) were synthesized by Umine (Shanghai, China). For transient overexpression of RdRp, BHK‐21 cells were transfected with negative control, MTase, or RdRp plasmids for 24 h using Lipofectamine 2000 transfection reagent (Thermo Fisher Scientific), following the manufacturer's instructions. The transfection efficiency of RdRp was determined by immunofluorescence and western blot analysis.

### 4.9 Identification the Binding Sites of PN‐1 to RdRp by LC‐MS

The Recombinant DENV‐2 RdRp protein was incubated with PN‐1 (10 µM) or DMSO at 4°C for 24 h. After adding loading buffer, protein samples were separated by 10% SDS‐PAGE and visualized by Coomassie blue staining. The bands in the gel corresponding to RdRp were excised, followed by in‐gel trypsin digestion and peptide extraction, following a previously reported protocol [47].

The peptide samples were analyzed using a nano‐HPLC system (Nano Elute, Bruker Daltonics, Billerica, MA, USA). The peptides dissolved in solvent A (H_2_O containing 0.1% formic acid) were loaded onto an analytical column (75 µm × 25 cm) and eluted with the following gradient: 2%–22% solvent B (acetonitrile containing 0.1% formic acid) for 45 min, 22%–37% B for 5 min, 37%–80% B for 5 min, and then 80% B for 5 min. Full‐scan MS spectra (*m*/*z* 100–1700) were acquired using a hybrid trapped ion mobility spectrometry quadrupole time‐of‐flight mass spectrometer (TIMS‐TOF Pro2; Bruker Daltonics). The MS data were analyzed using Fragpipe (version 19.0) for protein/peptide identification, as previously reported [48].

### 4.10 HDX‐MS

Recombinant RdRp was preincubated with PN‐1 (10 µM) or equivalent amounts of DMSO for 12 h at 4°C. Hydrogen exchange was initiated by the addition of 50 µL D_2_O solution (20 mM HEPES, 150 mM NaCl, pH 7.4) to 5 µL RdRp protein and PN‐1 samples. After incubation at various time points (10 s, 1 min, 5 min, 30 min, 2 h, and 9 h), the exchange reactions were quenched by the addition of cold quenching buffer (4 M guanidine hydrochloride, 0.5 M TCEP, 200 mM citric acid, pH 2.3). Samples were injected and subjected to pepsin digestion using an immobilized pepsin column (2.1 × 30 mm, NovaBioAssays, Woburn, MA, USA) and then trapped and desalted using an AcclaimTM PepMapTM 100 C18 HPLC column (1 × 5 mm, 5 µm, Thermo Fisher Scientific). The desalted peptides were eluted and separated on a C18 analytical column (1 × 50 mm, 1.9 µm, Thermo Fisher Scientific) with a linear gradient starting with 94% solvent A (water containing 0.1% formic acid)/6% solvent B (acetonitrile containing 0.1% formic acid) and increasing to 10% solvent A/90% solvent B at a flow rate of 45 µL/min within 15 min. Mass spectrometric data were obtained using a Fusion Orbitrap mass spectrometer (Thermo Fisher Scientific) operated in positive mode with a scan range of 300–1,500 *m*/*z*. The acquired data were processed with HDExaminer (Sierra Analytics, San Rafael, CA, USA) for the quantification of deuteration incorporation.

### 4.11 Time‐of‐Drug‐Addition Assay

BHK‐21 cells were cultured in 96‐well plates at a density of 8 × 10^3^ per well. For the prophylactic assay, BHK‐21 cells were pretreated with the indicated concentrations of PN‐1 for 5 h and inoculated with 200 plaque‐forming units (PFU)/mL DENV‐2 for 1 h at 37°C. The inocula were then replaced with RPMI‐1640 medium containing 2% FBS. For virus binding assay, cells were inoculated with DENV‐2 with or without PN‐1 and incubated at 4°C for 1 h to permit viral adsorption (without internalization). After removing the inoculum, cells were washed twice with PBS, replenished with RPMI‐1640 containing 2% FBS, and cultured at 37°C/5% CO_2_. For virus entry assay, cells were pre‐adsorbed with DENV‐2 (200 PFU/mL) at 4°C for 1 h. The viral suspension was then replaced with PN‐1‐supplemented RPMI‐1640 and incubated at 37°C for 1 h. Finally, the medium was refreshed with RPMI‐1640 containing 2% FBS for culture at 37°C/5% CO_2_. For virus intracellular replication assay, cells were exposed to 200 PFU/mL DENV‐2 for 1 h at 37°C. The infected cells were washed twice with PBS and replenished with RPMI‐1640 medium containing 2% FBS and different concentrations of PN‐1. After incubation for 4 days, 10 µL of the CCK‐8 solution was added to each well and incubated for 2 h at 37°C. Cell viability was determined by measuring absorbance at 450 nm using a microplate reader (Thermo Fisher Scientific).

### 4.12 Plaque Assay

BHK‐21 cells grown in 6‐well plates were infected with 200 PFU/mL DENV‐2 at 37°C and then treated with PN‐1 (2.5, 5, and 10 µM) or RV (20 µM) for 48 h. The supernatant containing the progeny virus was then transferred to a six‐well plate. After virus adsorption for 1 h, the infected cells were cultured in RPMI‐1640 medium containing 2% FBS and 1.2% methylcellulose (Sigma‐Aldrich, St Louis, MO, USA) for another 7 days. Finally, cells were washed with PBS, fixed with 4% paraformaldehyde, and stained with 2% crystal violet for 15 min to visualize plaque formation.

### 4.13 Mice

All animal experimental procedures were approved by the Experimental Animal Ethics Committee of the Guangzhou University of Chinese Medicine (approval numbers: 20220526001 and 20230330002 for ICR suckling and AG129 mice, respectively).

All animals were housed at the biosafety level II facility of Guangzhou University of Chinese Medicine under a 12‐h light/dark cycle. Six‐day‐old ICR suckling mice were purchased from SiPeiFu Biotechnology Co. Ltd. (Beijing, China). AG129 mice were provided by Prof. Jianping Zuo (Shanghai Institute of Materia Medica, Chinese Academy of Sciences), who was one of the authors of this study. Male and female AG129 mice, aged between 6 and 8 weeks, were selected.

### 4.14 DENV Infection Mice Model

All mouse infection experiments were conducted using the DENV‐2 NGC strain. PN‐1 used in the animal experiments was dissolved in vehicle (5% DMSO, 5% Tween 80, and 90% saline).

ICR suckling mice (n = 10 per group) were infected by a combination of intracranial and intraperitoneal inoculations. Each mouse first received an intracranial injection of 20 µL of DENV‐2 (400 PFU/mL) and was then administered an intraperitoneal injection of 50 µL of DENV‐2 (4 × 10^5^ PFU/mL). After infection, the mice were intraperitoneally administered PN‐1 (1, 2, and 4 mg/kg) daily. Body weights, clinical scores, and survival rates were monitored daily. The clinical symptoms were graded as previously reported: 0, no symptoms; 1, slight weight loss and ruffled hair; 2, retardation; 3, dyspraxia; 4, paralysis; 5, death [49]. The mice were anesthetized, and the main organs were collected for subsequent analysis.

AG129 mice were randomly divided into three groups (*n* = 8 per group): (1) control group, (2) DENV‐2 group, and (3) DENV‐2 + PN‐1 group. Subsequently, the mice were intraperitoneally inoculated with 200 µL DENV‐2 (4 × 10^5^ PFU/mL). The PN‐1 treatment group was intraperitoneally administered 10 mg/kg PN‐1 daily for nine days. The body weight and survival rate were recorded every day. On days 3, 5, and 7 post‐inoculations, blood was collected by tail clipping to quantify viral RNA copy numbers. Finally, liver, colon, and small intestine tissues were harvested for histopathological examination.

### 4.15 Histopathological Analysis and Immunohistochemistry

The tissues were fixed with 4% paraformaldehyde solution for over 24 h, dehydrated in a series of alcohol gradients and embedded in paraffin. Tissue sections (4 µm thick) were stained with H&E and visualized under an IX 53 light microscope (Olympus, Tokyo, Japan). Immunohistochemistry was performed as described previously [50]. Primary E antibody (Cat# GTX127277, GeneTex, Alton Pkwy Irvine, CA, USA; 1:400) was added to the tissue sections at 4°C overnight, and secondary antibody was further incubated for 30 min at RT.

### 4.16 Statistical Analysis

GraphPad Prism Software (version 8.0, San Diego, CA, USA) was used for statistical analysis. Data are expressed as mean ± standard deviation (SD) of at least three independent experiments. Statistical significance was performed by Student's *t*‐test between two different groups or by one‐way ANOVA in multiple groups. Kaplan–Meier survival analysis was used to compare the differences in survival probabilities in different groups. *p* < 0.05 was considered as statistically significant.

## Author Contributions

Linzhong Yu, Junshan Liu, and Xuemei He conceived and supervised the study. Xuemei He, Lifang Zou, and Jingtao Yu wrote the manuscript. Xuemei He, Lifang Zou, Jingtao Yu, and Tangjia Yang performed the experiments and analyzed the data. Zibin Lu, Huihui Cao, Wen Li, and Bing Chen participated in conducting experiments and provided intellectual expertise. Wei Zhao provided essential reagents and analyzed the experiments; Jianping Zuo provided AG129 mice. All authors have read and approved the final manuscript.

## Ethics Statement

All animal experimental procedures were approved by the Experimental Animal Ethics Committee of the Guangzhou University of Chinese Medicine (approval numbers: 20220526001 and 20230330002 for ICR suckling and AG129 mice, respectively).

## Conflicts of Interest

The authors declare no conflicts of interest.

## Supporting information




**Table S1**. Primers and probes for qRT‐PCR assay.
**Figure S1** The chemical structures of fourteen dammarane‐type triterpenoid saponins.
**Figure S2** The purity of PN‐1 determined by HPLC analysis.
**Figure S3** PN‐1 directly targets NS5. (A) Quantification of NS5 protein levels after heat challenge (40‐75°C) in the presence and absence of PN‐1. (B, C) Quantification of NS5 protein levels after pronase digestion. ^*^ *P* <0.05, ^**^ *P* <0.01, ^***^ *P* <0.001.
**Figure S4** The MS (A) and ^1^H NMR (B) analysis of Bio‐PN‐1.
**Figure S5** The cytotoxicity of Bio‐PN‐1 in BHK‐21 cells. Cells were treated with different concentrations of Bio‐PN‐1 for 48 h and then examined by MTT assay. ^***^ *p* < 0.001 versus control model.
**Figure S6** (A, B) Quantification of NS5 protein levels from pull down assays. Data from Western blot densitometry. ^***^ *p* < 0.001.
**Figure S7** PN‐1 interacts with NS5 protein. The binding mode of PN‐1 with DENV‐2 NS5 using molecular docking.
**Figure S8** The transfection efficiency of MTase and RdRp plasmids was detected by immunofluorescence assay (A) and Western blot analysis (B).
**Figure S9** PN‐1 directly targets RdRp. (A) Quantification of RdRp protein levels after heat challenge (40‐75°C) in the presence and absence of PN‐1. (B, C) Quantification of RdRp protein levels after pronase digestion. Data from Western blot densitometry. ^***^ *P* <0.001.
**Figure S10** MTase is not the target of PN‐1 against DENV‐2. (A, B) The resistance of MTase to protease was assessed by DARTS assays. (C, D) The binding mode of PN‐1 with DENV‐2 MTase.
**Figure S11** PN‐1 directly targets RdRp. (A, B) Quantification of RdRp protein levels from pull down assays using RdRp transfected cell lysates. (C, D) Quantification of RdRp protein levels from pull down assays using recombinant RdRp. (E) Quantification of RdRp and its subdomains fingers, palm and thumb protein levels from pull down assays. Data from Western blot densitometry. ^***^ *p* < 0.001.
**Figure S12** (A, B) The MS/MS analysis of PN‐1.
**Figure S13** PN‐1 covalently modified RdRp at residues M387 (A, B) and E479 (C, D). A, C: DMSO control; B, D: PN‐1 treated. Recombinant RdRp protein was incubated with DMSO (left panels) or PN‐1 (right panels) at 4°C for 24 h. Green fragment ion peaks correspond to b‐ions; orange fragment ion peaks represent y‐ions.
**Figure S14** The transfection efficiency of E255A, M387A, E479A and A507G plasmids was detected by immunofluorescence assay (A) and Western blot analysis (B).
**Figure S15** Quantification of RdRp protein levels from pull down assays using WT or mutated RdRp plasmids transfected cell lysates. Data from Western blot densitometry. ^**^ *p* < 0.01, ^***^ *p* < 0.001.
**Figure S16** The peptide coverage of RdRp in hydrogen‐deuterium exchange‐mass spectrometry.
**Figure S17** The dynamic changes of RdRp after PN‐1 treatment using hydrogen‐deuterium exchange‐mass spectrometry. (A) Heatmap of PN‐1 and RdRp at all hydrogen deuterium exchange time points. (B) The peptides with higher levels of hydrogen–deuterium exchange after PN‐1 treatment have been marked in blue.
**Figure S18** PN‐1 does not inhibit DENV‐2 attachment/entry in BHK‐21 cells. (A) Relative cell viability of BHK‐21 cells under PN‐1 treatment. PN‐1 had no obvious effect on DENV‐induced cell death when administered pre‐infection (B), at the viral adsorption phase (C), or at the entry phase (D). The cell viability of BHK‐21 cells was detected by CCK‐8 assay. ^***^ *p* < 0.001.
**Figure S19** PN‐1 suppresses DENV‐2 protein expression in BHK‐21 cells. E (A) and NS1 (B) protein levels in cells infected with DENV‐2 and treated with PN‐1. Quantified by Western blot densitometry. ^***^ *p* < 0.001.
**Figure S20** Effects of PN‐1 on the cell viability of Huh7, HepG2, 293T and Vero cells assessed by MTT assay.
**Figure S21** Safety assessment of PN‐1 in ICR suckling mice. (A) Body weight changes during 6‐day treatment with PN‐1 (1, 2, 4 mg/kg/day, i.p.) or vehicle. (B) Representative H&E‐stained sections of heart, liver, spleen, lung and kidney tissues. No significant histopathological alterations observed compared to untreated controls.
**Figure S22** PN‐1 suppresses DENV‐2 replication in mouse brain. (A‐B) Quantification of viral E (A) and NS1 (B) protein levels in brain tissues of ICR suckling mice infected with DENV‐2 and treated with PN‐1. Data from Western blot densitometry. ^***^ *p* < 0.001.
**Figure S23** Histopathological analysis of heart, liver, spleen, lung and kidney tissues from DENV‐2‐infected ICR suckling mice. Tissues harvested under anesthesia show no significant alterations compared to uninfected controls.

## Data Availability

The data included in this study are available upon request from the corresponding author.
