# Supplementary material for: Discovery of a Novel Non‐Nucleoside Inhibitor of RNA‐Dependent RNA Polymerase Against Dengue Virus
Source: MedComm (2020). 2025 Nov 29;6(12):e70514. doi: 10.1002/mco2.70514 (PMC12664905; doi:10.1002/mco2.70514)
Supplement: Supplementary file 1 — Table S1. Primers and probes for qRT‐PCR assay. Figure S1 The chemical structures of fourteen dammarane‐type triterpenoid saponins. Figure S2 The purity of PN‐1 determined by HPLC analysis. Figure S3 PN‐1 directly targets NS5. (A) Quantification of NS5 protein levels after heat challenge (40‐75°C) in the presence and absence of PN‐1. (B, C) Quantification of NS5 protein levels after pronase digestion. * P <0.05, ** P <0.01, *** P <0.001. Figure S4 The MS (A) and 1H NMR (B) analysis of Bio‐PN‐1. Figure S5 The cytotoxicity of Bio‐PN‐1 in BHK‐21 cells. Cells were treated with different concentrations of Bio‐PN‐1 for 48 h and then examined by MTT assay. *** p < 0.001 versus control model. Figure S6 (A, B) Quantification of NS5 protein levels from pull down assays. Data from Western blot densitometry. *** p < 0.001. Figure S7 PN‐1 interacts with NS5 protein. The binding mode of PN‐1 with DENV‐2 NS5 using molecular docking. Figure S8 The transfection efficiency of MTase and RdRp plasmids was detected by immunofluorescence assay (A) and Western blot analysis (B). Figure S9 PN‐1 directly targets RdRp. (A) Quantification of RdRp protein levels after heat challenge (40‐75°C) in the presence and absence of PN‐1. (B, C) Quantification of RdRp protein levels after pronase digestion. Data from Western blot densitometry. *** P <0.001. Figure S10 MTase is not the target of PN‐1 against DENV‐2. (A, B) The resistance of MTase to protease was assessed by DARTS assays. (C, D) The binding mode of PN‐1 with DENV‐2 MTase. Figure S11 PN‐1 directly targets RdRp. (A, B) Quantification of RdRp protein levels from pull down assays using RdRp transfected cell lysates. (C, D) Quantification of RdRp protein levels from pull down assays using recombinant RdRp. (E) Quantification of RdRp and its subdomains fingers, palm and thumb protein levels from pull down assays. Data from Western blot densitometry. *** p < 0.001. Figure S12 (A, B) The MS/MS analysis of PN‐1. Figure S13 PN‐1 covalently modified [file MCO2-6-e70514-s001.docx]

**Discovery of a Novel Non-nucleoside Inhibitor of RNA-dependent RNA Polymerase against Dengue Virus**

Xuemei He^1,#^, Lifang Zou^1,#^, Jingtao Yu^1,#^, Tangjia Yang^1^, Zibin Lu^1^, Huihui Cao^1^, Wen Li^2^, Bing Chen^1^, Wei Zhao^3^, Jianping Zuo^4^, Linzhong Yu^1,*^, Junshan Liu^1,*^

^1^School of Traditional Chinese Medicine, Guangdong Provincial Key Laboratory of Chinese Medicine Pharmaceutics, Guangdong Basic Research Center of Excellence for Integrated Traditional and Western Medicine for Qingzhi Diseases, Southern Medical University, Guangzhou, China

^2^Analytical and Testing Center, Jinan University, Guangzhou, China

^3^Guangdong Provincial Key Laboratory of Tropical Disease Research, School of Public Health, Southern Medical University, Guangzhou, China

^4^Laboratory of Immunopharmacology, State Key Laboratory of Drug Research, Shanghai Institute of Materia Medica, Chinese Academy of Sciences, Shanghai, China

^*^Correspondence: Junshan Liu (liujunshan@smu.edu.cn); Linzhong Yu (yulzh@smu.edu.cn).

^#^These authors made equal contributions to this work.

**Supplementary materials**

**Supplementary methods**

- 1. **Chemicals**

Fourteen dammarane-type triterpenoid saponins (Figure S1) from the roots of *P. notoginseng* were isolated and identified by Prof. Chunlin Fan (the College of Pharmacy, Jinan University) as previously described [1]. The purity of PN-1 determined by HPLC analysis was more than 99% (Figure S2).

- 1. **Cell Culture**

Baby hamster kidney fibroblast cells (BHK-21) and *Aedes albopictus* cells (C6/36) were cultured in RPMI-1640 medium containing 10% fetal bovine serum (FBS, ExCell Bio, Suzhou, China). African green monkey kidney cells (Vero), human embryonic kidney cells (293T), human hepatocellular carcinoma Huh7 and HepG2 cells were grown in DMEM containing 10% FBS. All cell lines were maintained in a humidified incubator at 37 °C and 5% CO_2_, except for C6/36, which was cultured at 28 °C and 5% CO_2_.

- 1. **Viruses**

The DENV-2 New Guinea C (NGC) strain was a gift from Professor Xingang Yao of Southern Medical University. DENV-1 strain Hawaii and DENV-3 strain H87 were stored in the Guangdong Provincial Key Laboratory of Tropical Disease Research (Guangdong, China). All viral stocks were propagated in C6/36 cells and harvested from culture supernatants until clear CPE was observed. The viral stocks were then stored at −80 °C, and virus titers were determined using the median tissue culture infectious dose (TCID_50_) assay.

- 1. **Molecular Docking**

The crystal structures of the DENV-2 enzymatic proteins NS5, MTase, and RdRp (PDB ID: 4V0R, 2P3L and 5K5M, respectively) were obtained from the RCSB Protein Data Bank. Molecular docking of PN-1 to the above proteins was performed using Maestro (Schrödinger, San Diego, CA, USA). The protein was prepared using the protein preparation wizard and water molecules were removed from the protein. Receptor grids were generated using Receptor Grid Generation. The docked structure and binding site of PN-1 into MTase and RdRp were displayed using PyMOL software (Schrӧdinger).

- 1. **MTT assay**

BHK-21, Huh7, HepG2, 293T, and Vero cells were seeded into 96-well culture plates at a density of 8 × 10³ cells *per* well and incubated at 37 °C for 24 h. Following treatment with various concentrations of PN-1 for either 48 or 96 h, MTT solution was added to each well and the plates were incubated for an additional 4 h. The supernatant was then carefully discarded, and 100 μL of dimethyl sulfoxide (DMSO) was added to dissolve the formazan crystals. The absorbance value was measured at 570 nm by the microplate reader (Thermo Fisher Scientific).

- 1. **Quantitative Real-Time PCR (qRT-PCR)**

Total RNA was extracted from cells or tissues using TRIzol reagent according to the manufacturer’s instructions. For mouse serum, viral RNA was extracted following the instructions of the E.Z.N.A.^®^Viral RNA Kit (Omega Bio-Tek, Norcross, GA, USA). PrimeScript™ RT Master Mix (Takara, Kyoto, Japan) was used to reverse transcribe the extracted RNA followed by PCR-amplification according to the instructions of the Bestar^R^ qPCR Master Mix (DBI Bioscience, Ludwigshafen, Germany). The primers and probes used in this study (Table S1) were synthesized by Invitrogen.

- 1. **Western Blot Analysis**

Harvested cells or chopped tissues were lysed with RIPA lysis buffer containing protease and phosphatase inhibitors for 15 min. Protein lysates were collected by centrifugation at 13,000 ×g at 4 °C for 15 min, followed by quantification using the BCA assay. The samples were then analyzed by 10% SDS-PAGE and transferred to polyvinylidene fluoride membranes. Membranes were blocked with 5% (*w*/*v*) skim milk for 1 h and incubated with primary antibodies at 4 °C overnight. The following primary antibodies were employed for detection: anti-DENV-2 E (Cat# GTX127277, GeneTex, Alton Pkwy Irvine, CA, USA; 1:3000), anti-DENV-2 NS1 (Cat# GTX124280, GeneTex, 1:3000), anti-DENV-2 NS5 (Cat# GTX629447, GeneTex, 1:2000), anti-FLAG M2 Antibody (Cat# 14793, Cell Signaling Technology, Danvers, MA, USA; 1:1000), anti-β-actin (Cat# 4970, Cell Signaling Technology; 1:1000). After washing with 0.05% Tween-20 buffer, the corresponding secondary antibodies were added and incubated for 2 h at 4 °C. Finally, the protein blots were visualized using the FluorChem E^TM^ system (ProteinSimple, San Francisco, CA, USA) based on an ECL kit.

- 1. **Immunofluorescence Assay**

Treated cells were fixed using 4% (*v*/*v*) formaldehyde for 10 min, followed by permeabilization with 0.1% (*v*/*v*) Triton-100 for 5 min. The cells were blocked with 5% (*w/v*) skim milk for 1 h and incubated with the primary antibodies at 4 °C overnight. The following primary antibodies were used for detection: anti-DENV-2 E (1:400), anti-DENV-2 NS1 (1:400), anti-DENV-2 NS5 (1:500), anti-FLAG M2 Antibody (1:50), Anti-Biotin (Cat# ab53494, Abcam, Cambridge, UK; 1:1000), Anti-dsRNA mAb J2 (Cat# 10010200, SCICONS, Szirák, Hungary; 1:1000). The cells were then incubated with Alexa Fluor 488-conjugated goat anti-rabbit secondary antibody (1:1000) or Alexa Fluor 555-conjugated anti-mouse secondary antibody (1:1000) at 4 °C for 1 h. Cell nuclei were stained with DAPI (1:500) for 5 min. Finally, the treated cells were observed under a confocal microscope (LSM800, Carl Zeiss, Oberkochen, Germany).

Reference

1. Y. R. Zheng, C. L. Fan, Y. Chen, et al., “Anti-inflammatory, anti-angiogenetic and antiviral activities of dammarane-type triterpenoid saponins from the roots of *Panax notoginseng*,” *Food Function* 13, no. 6 (2022): 3590-3602.

**Table S1.** Primers and probes for qRT-PCR assay.

| Gene | Primer and probe sequences |
| --- | --- |
| DENV1-NS1 | Forward: 5’-GATGAGATCCAG ATGGAGTAGAAAG-3’ |
|  | Reverse: 5’-TCAGTTGTCCCATTATAAGAAGGAG-3’ |
|  | Probe: 5’-FAM-ACAGCCAGTGTTCCAGTCATCAGCA-BHQ1-3’ |
| DENV2-E | Forward: 5’-CAGTCGGAAATGACACAG-3’ |
|  | Reverse: 5’-GCAACACCATCTCATTGA-3’ |
|  | Probe: 5’-FAM-AAGTAACACCACAGAGTTCCATCACA-BHQ1-3’ |
| DENV2-NS1 | Forward: 5’-CTTGAGATGGACTTTGATTTCTGC-3’ |
|  | Reverse: 5’-CTCTTCTTTCTCTTTCAAT GGTCTG-3’ |
|  | Probe: 5’-FAM-ACTCATAACAGAATGGTGCTGCCGATC-BHQ1-3’ |
| DENV3-NS5 | Forward: 5’-CAAAACTTCAATGGTTYGTGGA-3’ |
|  | Reverse: 5’-TGTGTATCCTCGCACTTCTGTRAC-3’ |
|  | Probe: 5’-FAM-AATATGACCAGCCTCCTCTTCCACAGCCY-BHQ1-3’ |

**Figure S1**


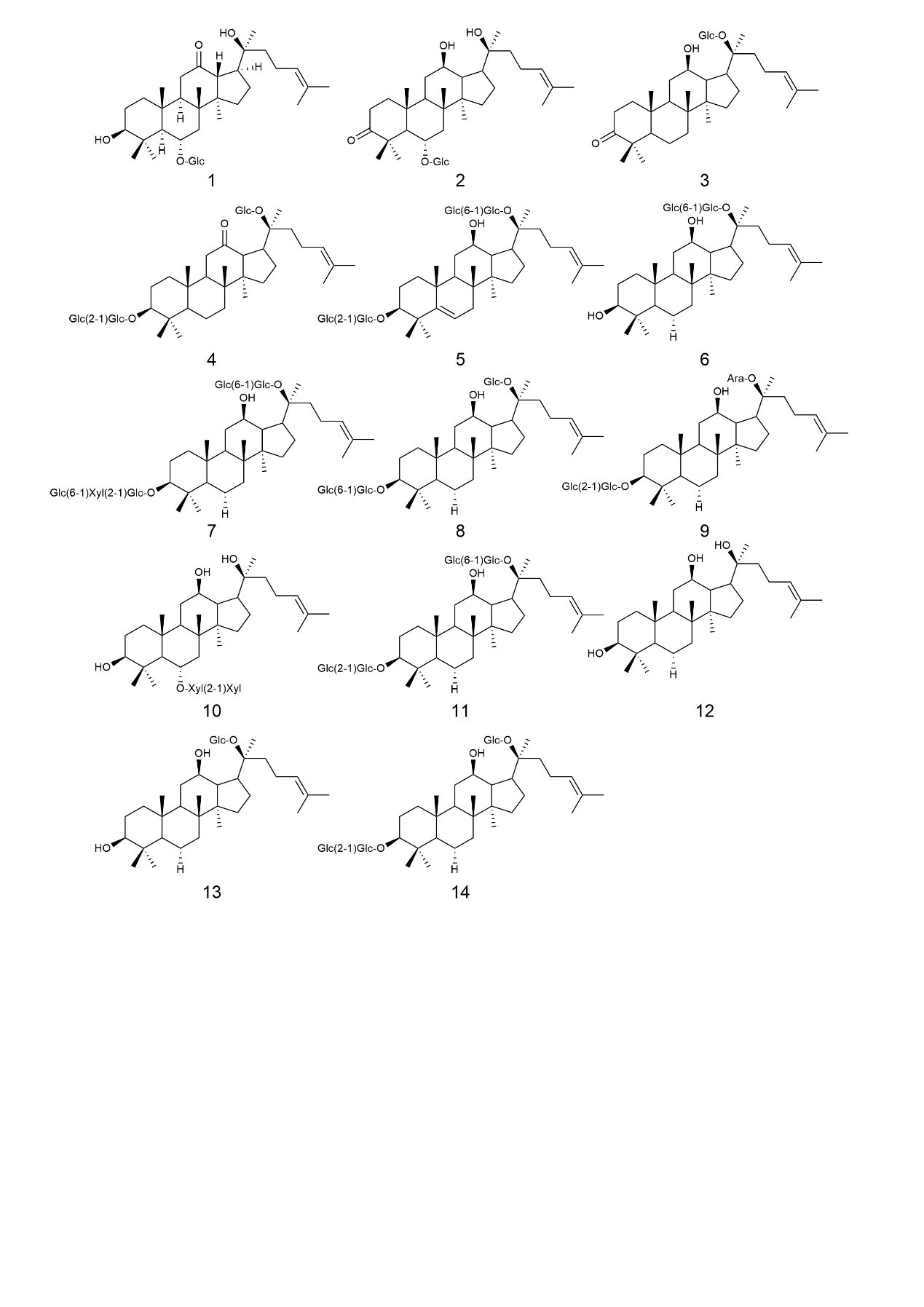


**Figure S1** The chemical structures of fourteen dammarane-type triterpenoid saponins.

**Figure S2**


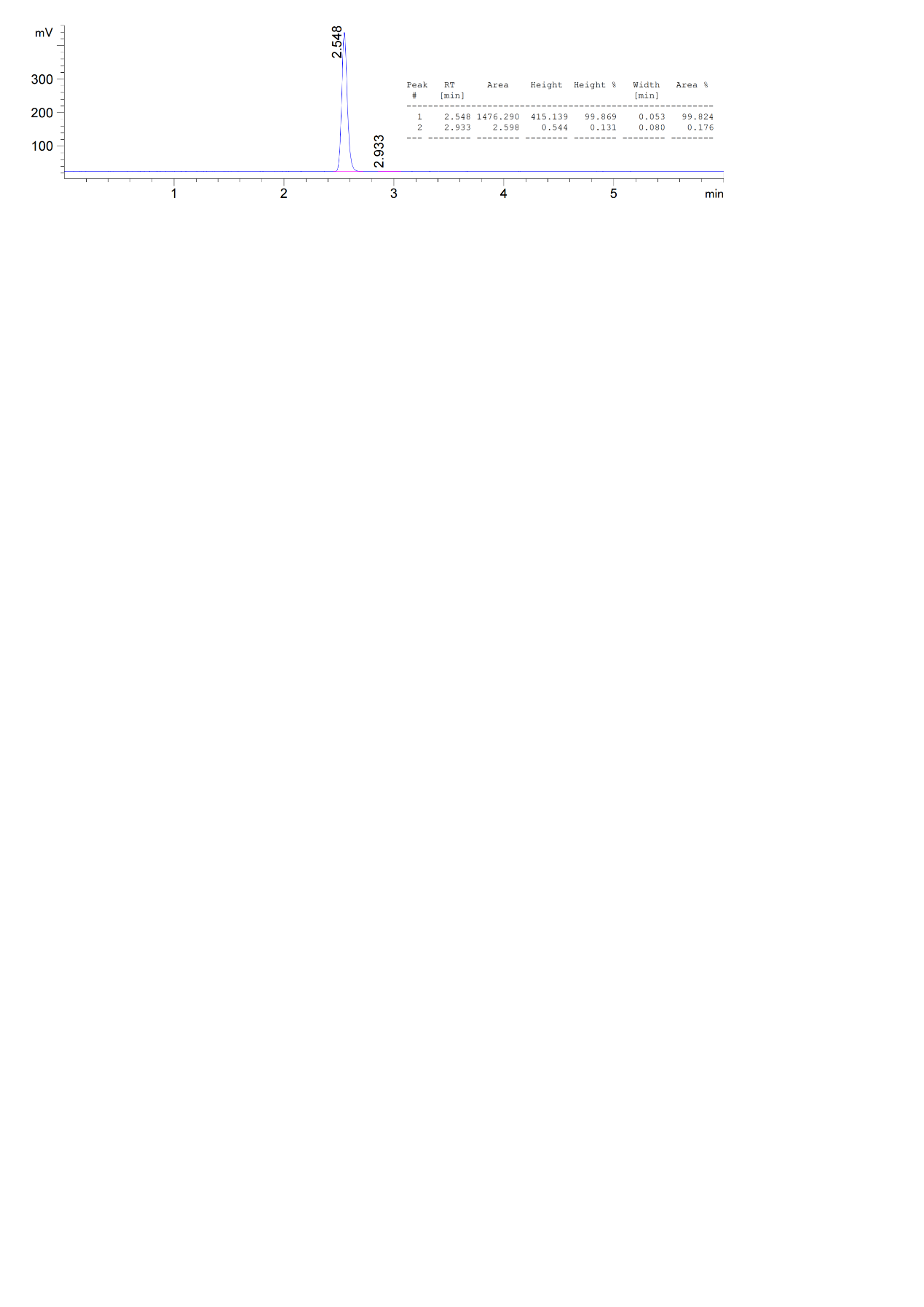


**Figure S2** The purity of PN-1 determined by HPLC analysis.

**Figure S3**


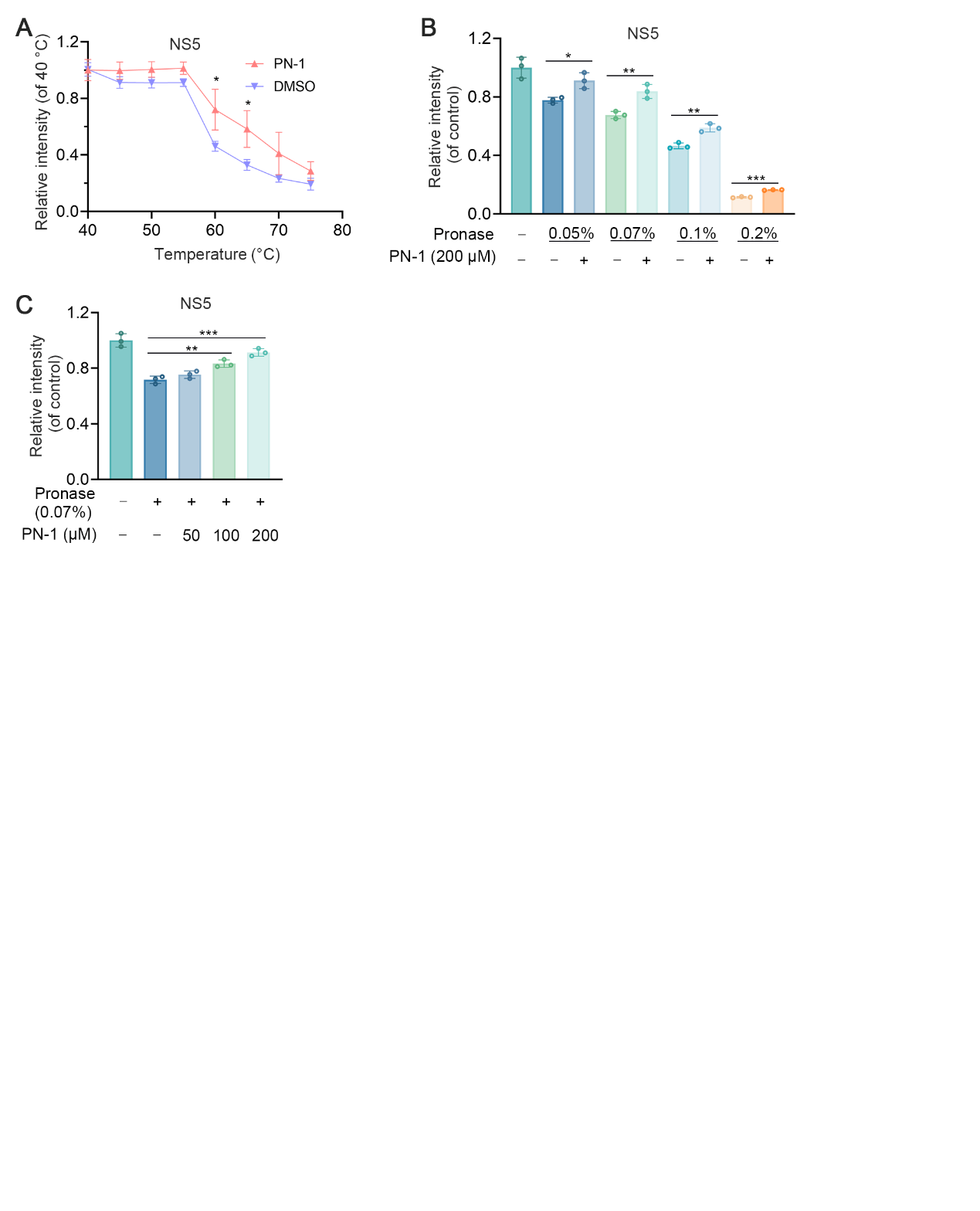


**Figure S3** PN-1 directly targets NS5. (A) Quantification of NS5 protein levels after heat challenge (40-75 °C) in the presence and absence of PN-1. (B, C) Quantification of NS5 protein levels after pronase digestion. ^*^*P* <0.05, ^**^*P* <0.01, ^***^*P* <0.001.

**Figure S4**


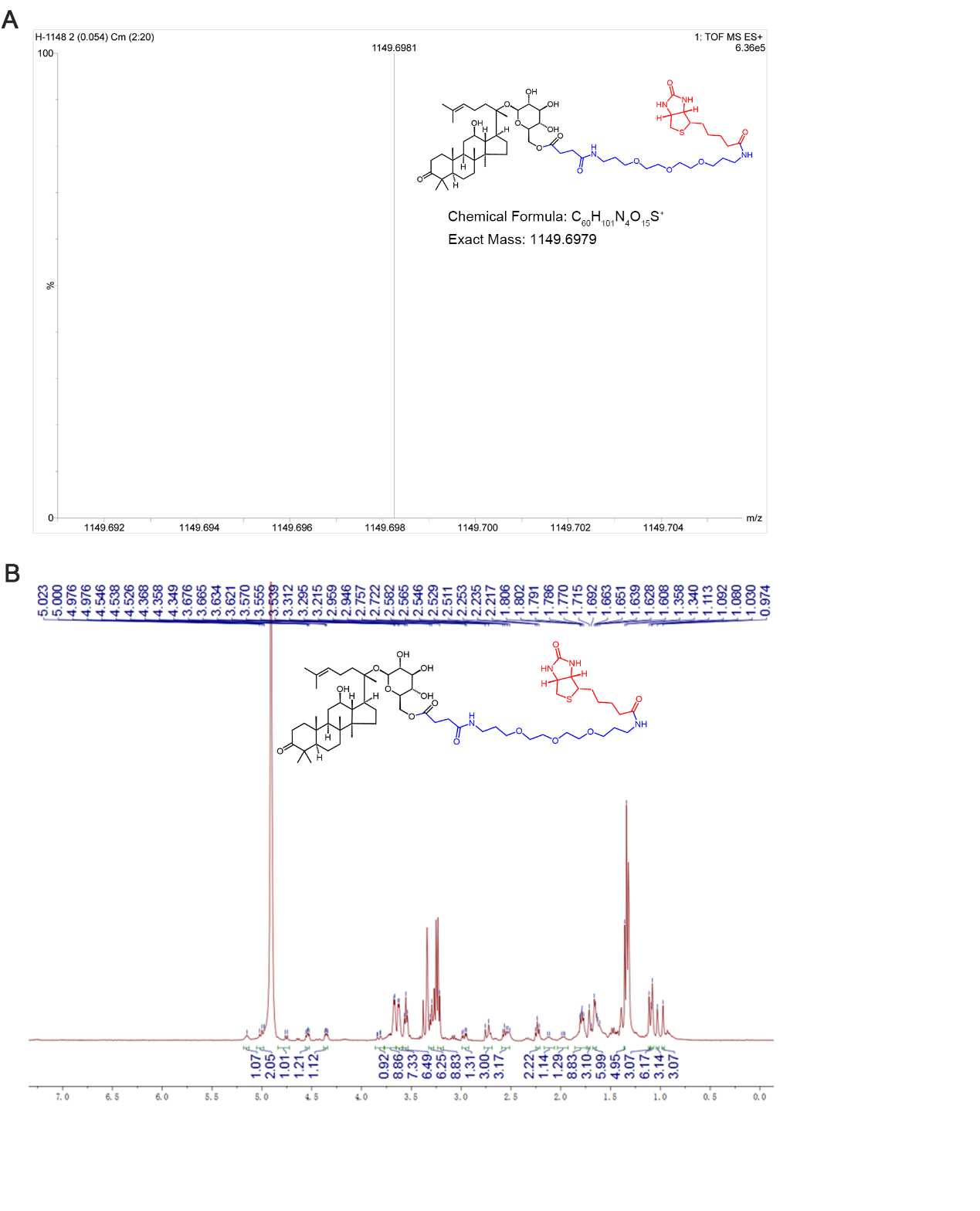


**Figure S4** The MS (A) and ^1^H NMR (B) analysis of Bio-PN-1.

**Figure S5**

**
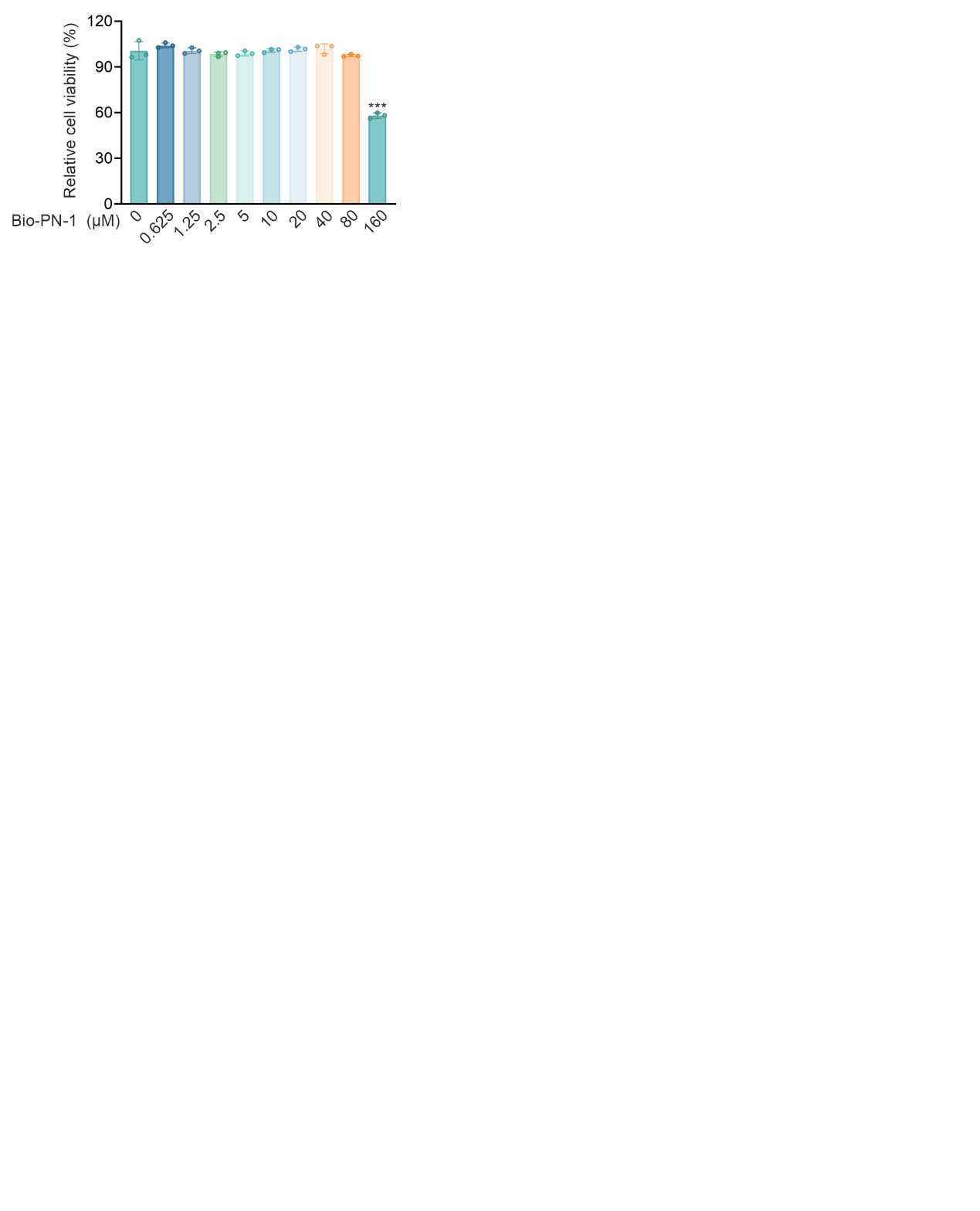
**

**Figure S5** The cytotoxicity of Bio-PN-1 in BHK-21 cells. Cells were treated with different concentrations of Bio-PN-1 for 48 h and then examined by MTT assay. ^***^*P* < 0.001 *versus* control model.

**Figure S6**


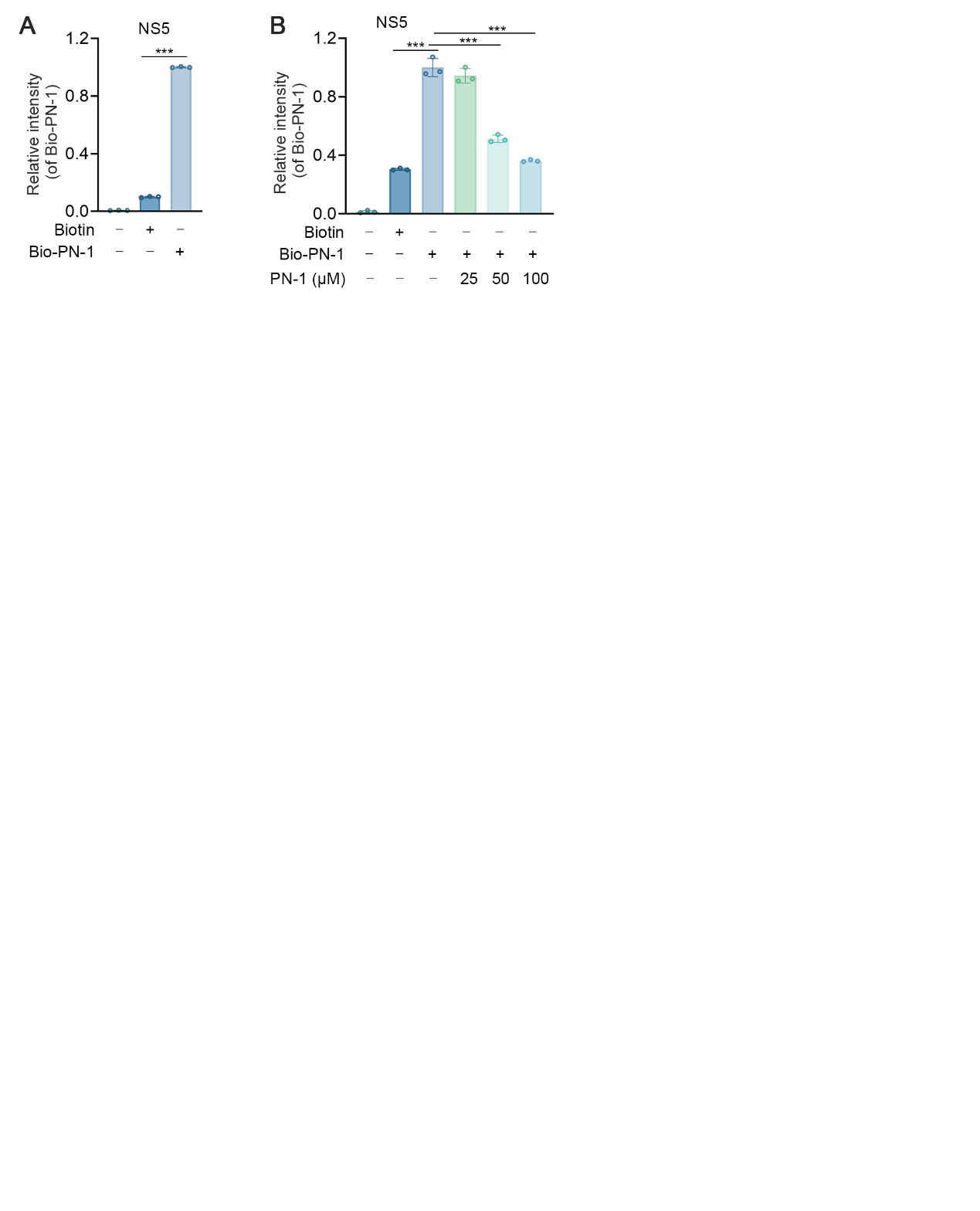


**Figure S6** (A, B) Quantification of NS5 protein levels from pull down assays. Data from Western blot densitometry. ^***^*P* < 0.001.

**Figure S7**


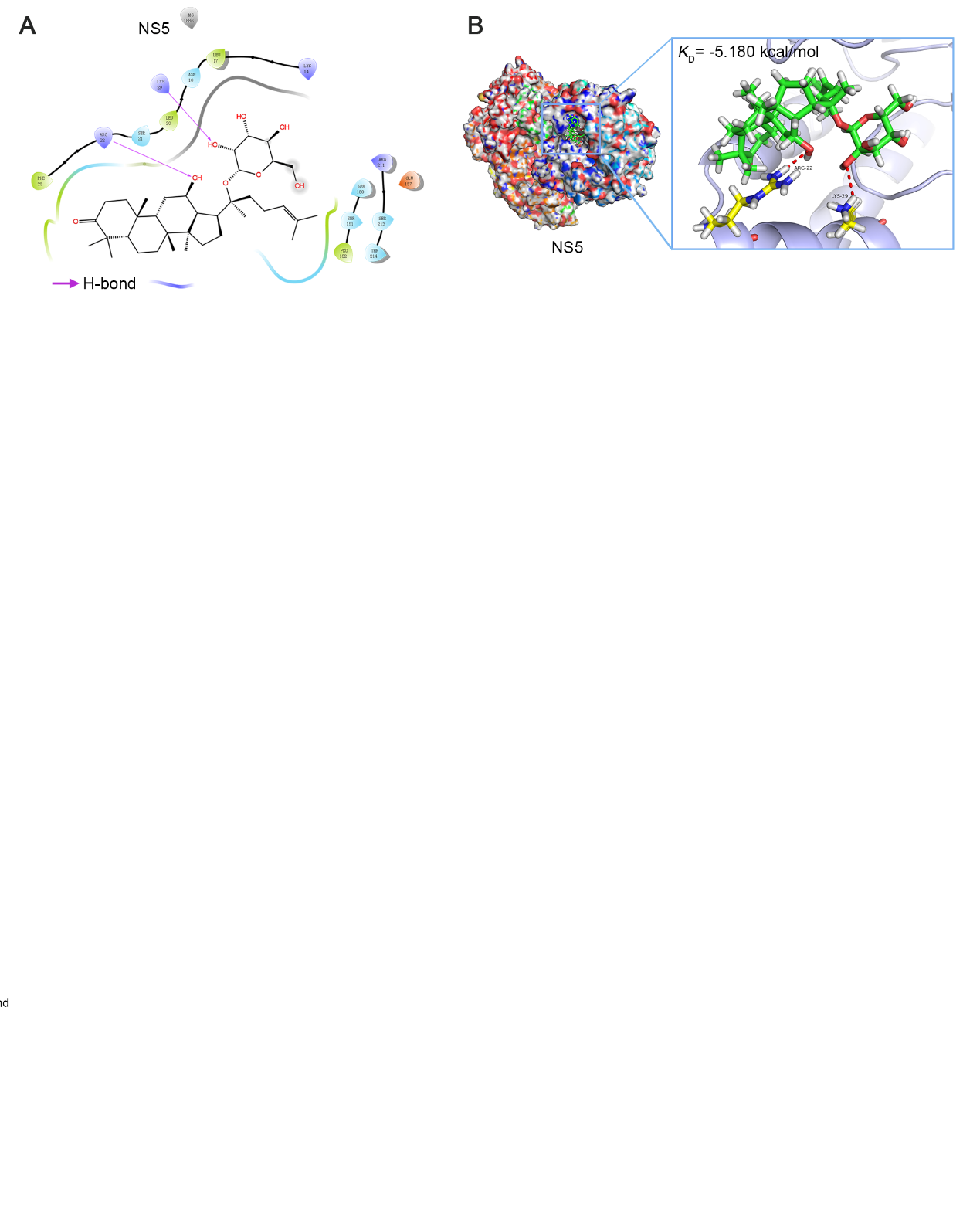


**Figure S7** PN-1 interacts with NS5 protein. The binding mode of PN-1 with DENV-2 NS5 using molecular docking.

**Figure S8**


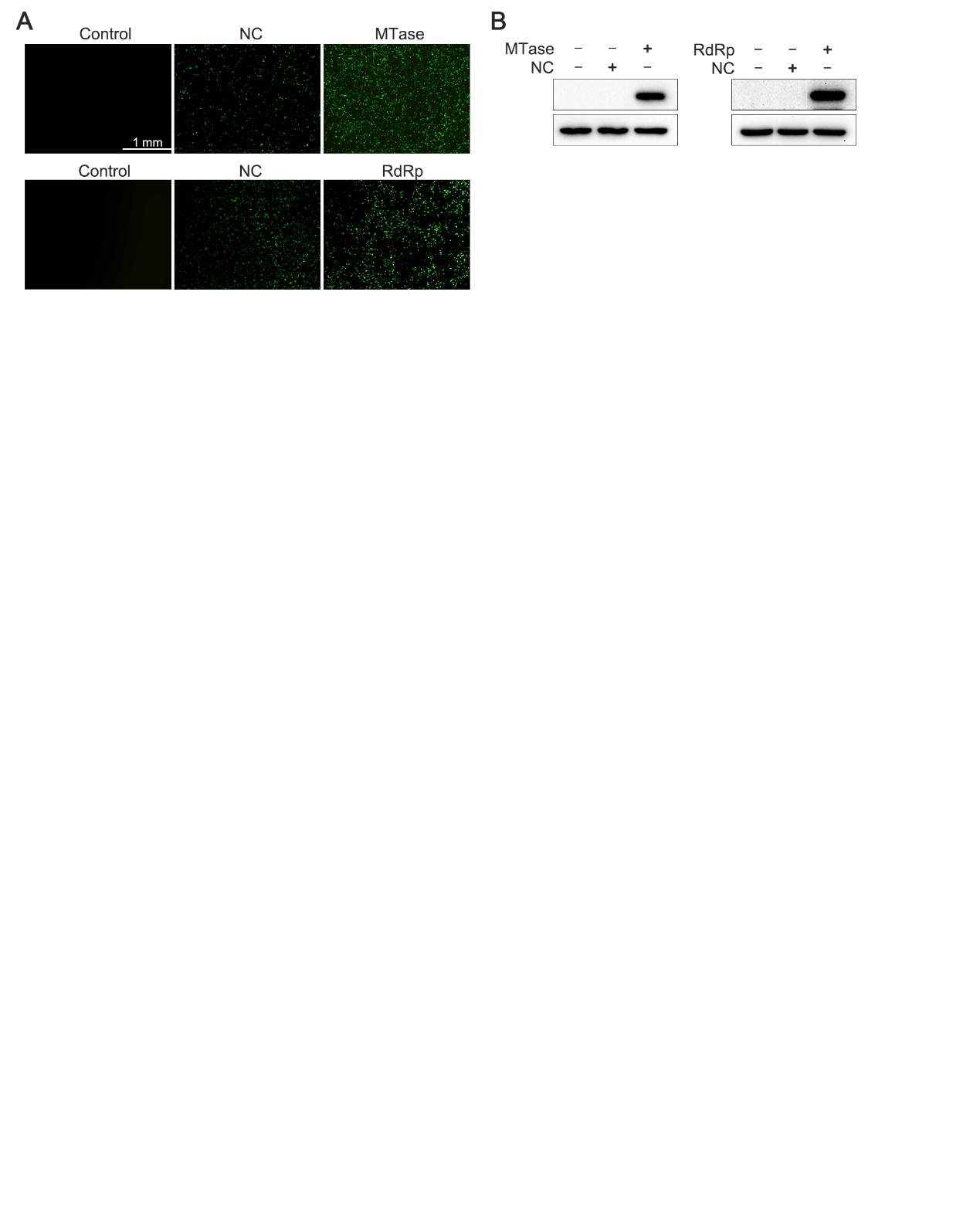


**Figure S8** The transfection efficiency of MTase and RdRp plasmids was detected by immunofluorescence assay (A) and Western blot analysis (B).

**Figure S9**


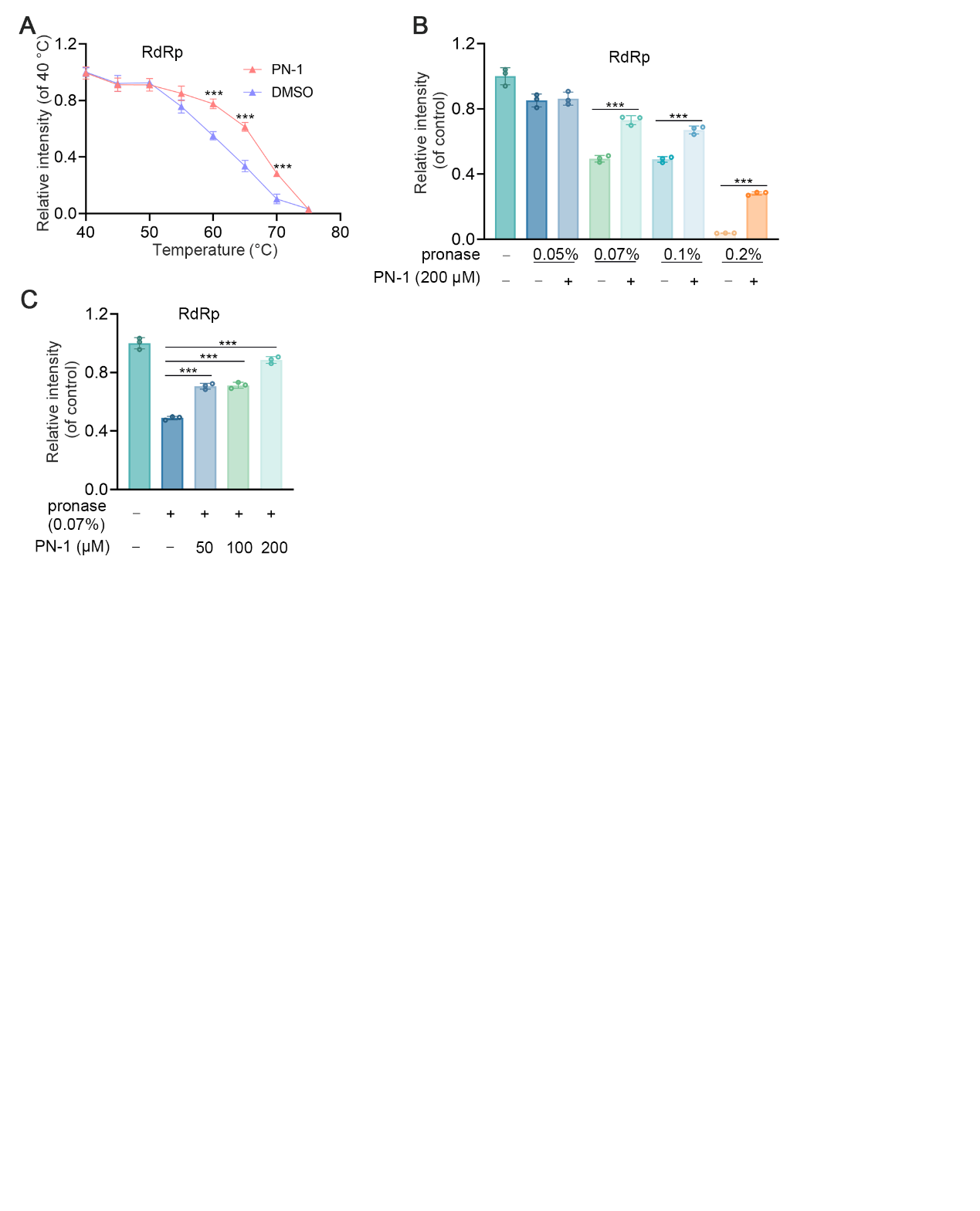


**Figure S9** PN-1 directly targets RdRp. (A) Quantification of RdRp protein levels after heat challenge (40-75 °C) in the presence and absence of PN-1. (B, C) Quantification of RdRp protein levels after pronase digestion. Data from Western blot densitometry. ^***^*P* <0.001.

**Figure S10**


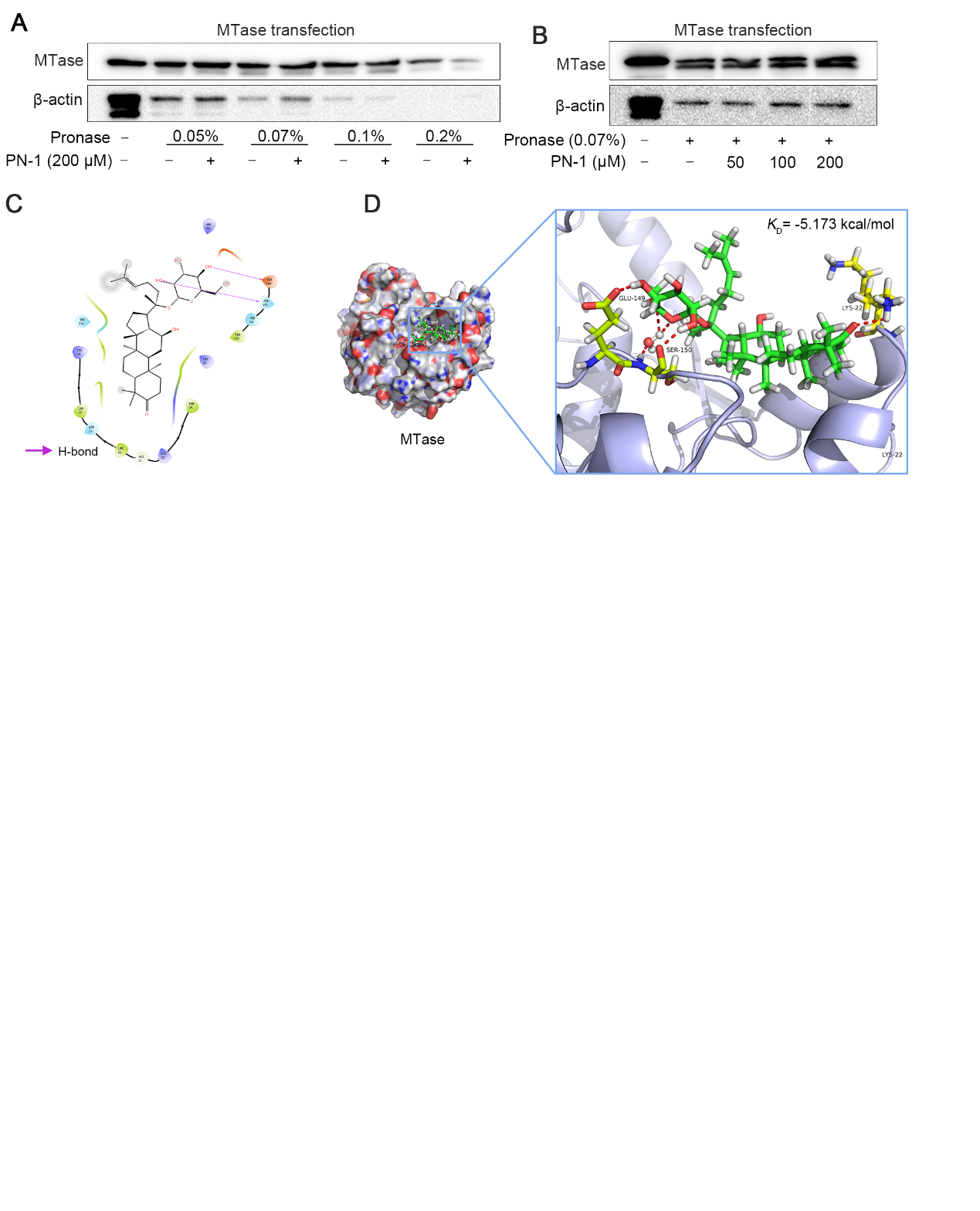


**Figure S10** MTase is not the target of PN-1 against DENV-2. (A, B) The resistance of MTase to protease was assessed by DARTS assays. (C, D) The binding mode of PN-1 with DENV-2 MTase.

**Figure S11**

**
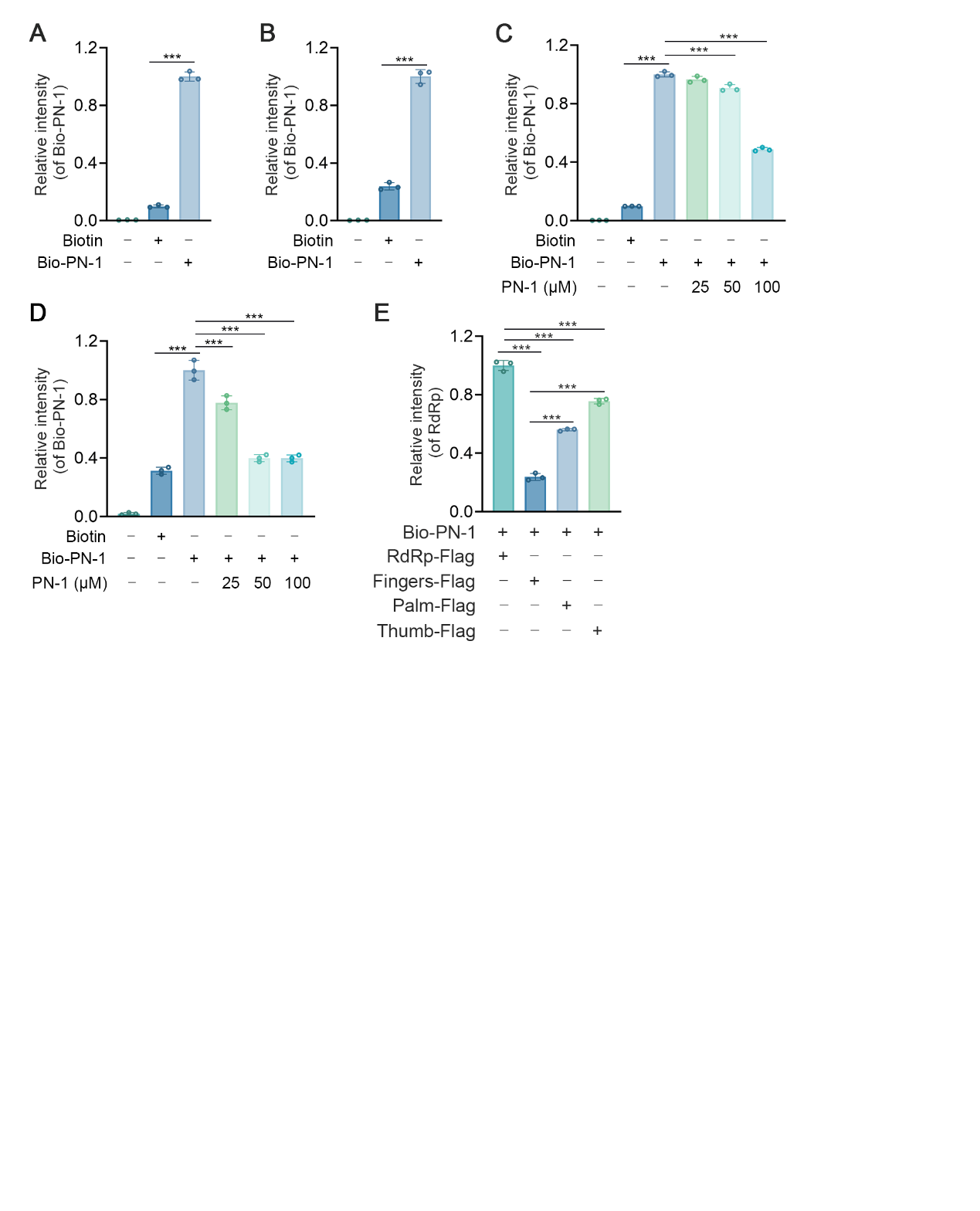
**

**Figure S11** PN-1 directly targets RdRp. (A, B) Quantification of RdRp protein levels from pull down assays using RdRp transfected cell lysates. (C, D) Quantification of RdRp protein levels from pull down assays using recombinant RdRp. (E) Quantification of RdRp and its subdomains fingers, palm and thumb protein levels from pull down assays. Data from Western blot densitometry. ^***^*P* < 0.001.

**Figure S12**


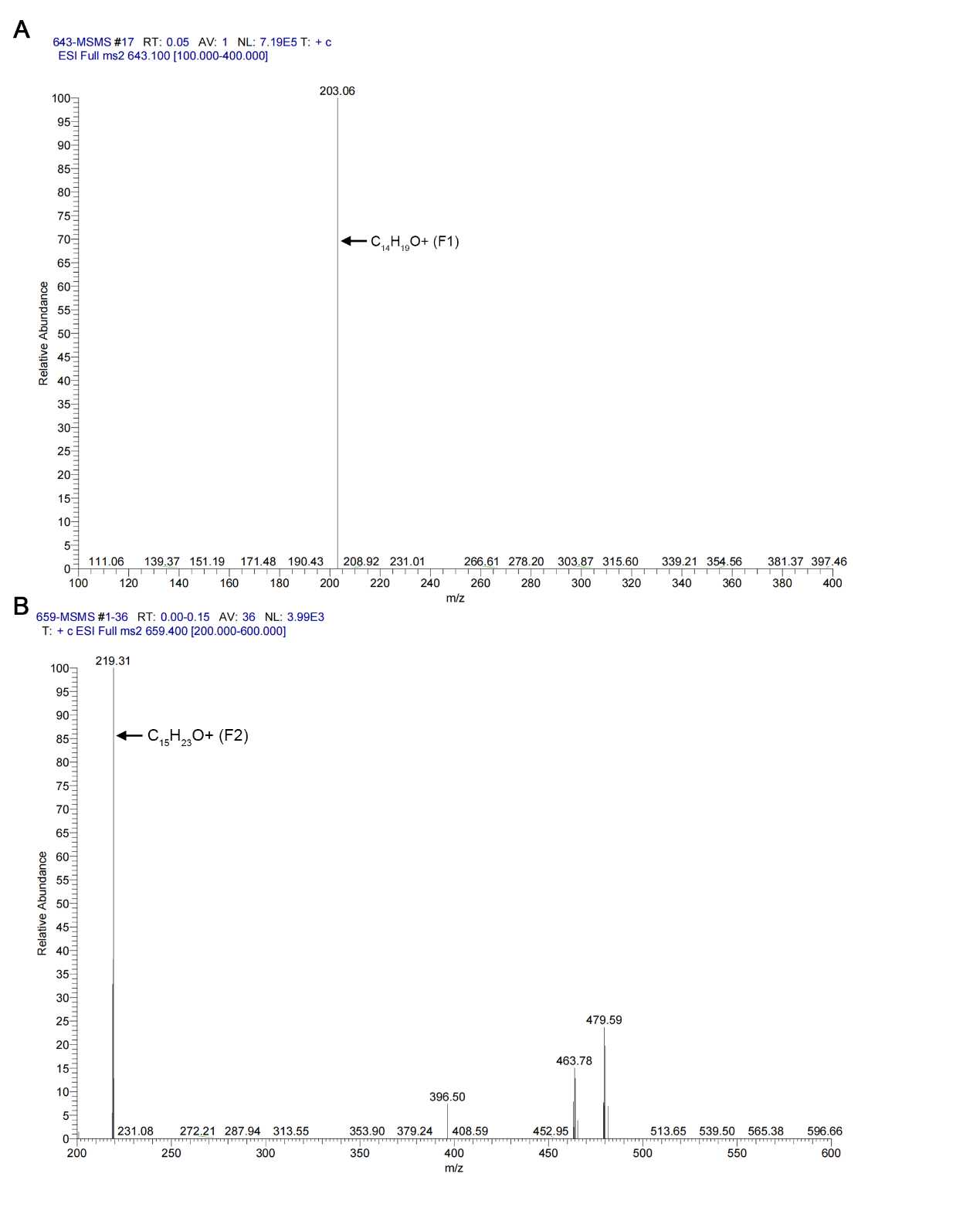


**Figure S12** (A, B) The MS/MS analysis of PN-1.

**Figure S13**


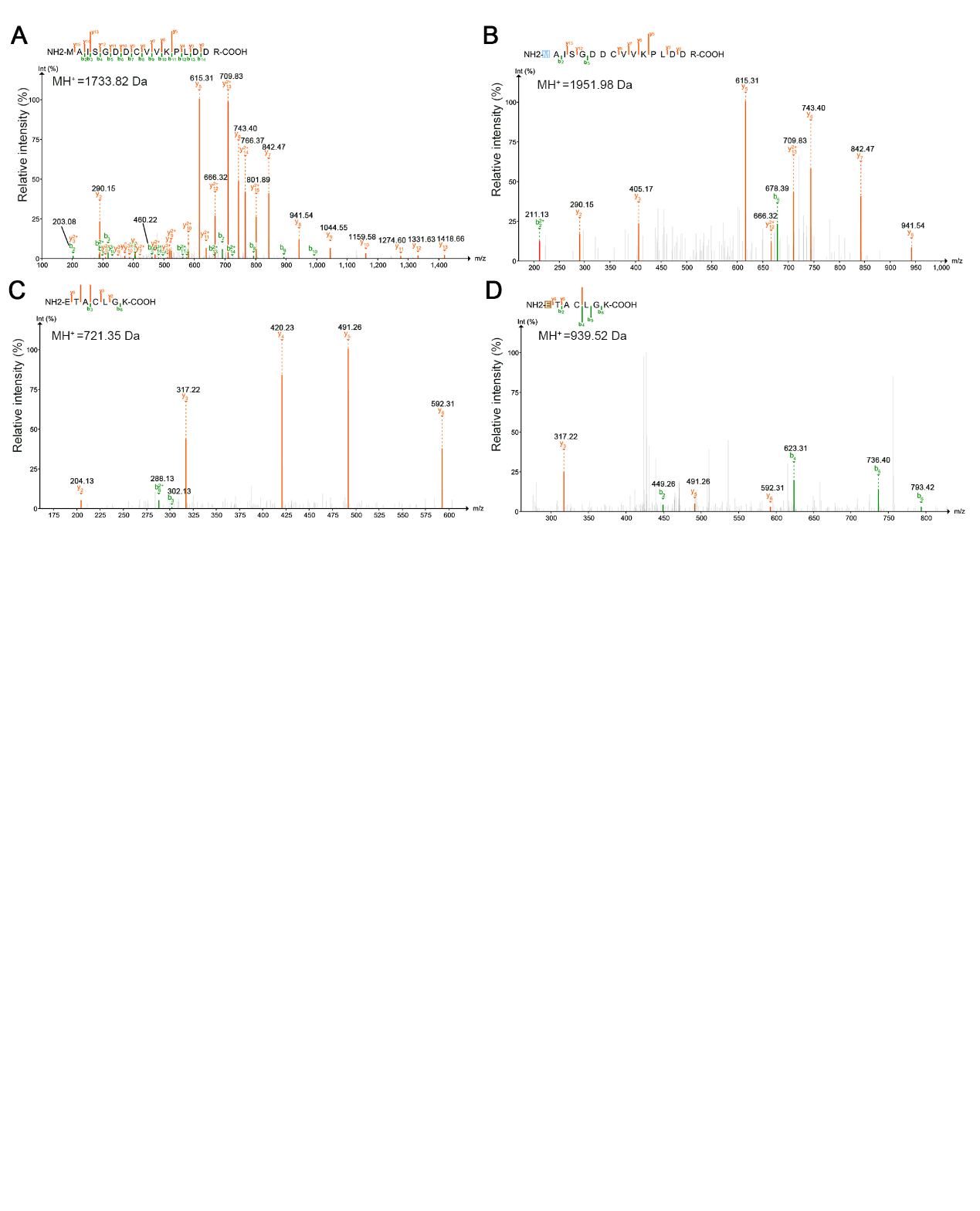


**Figure S13** PN-1 covalently modified RdRp at residues M387 (A, B) and E479 (C, D). A, C: DMSO control; B, D: PN-1 treated. Recombinant RdRp protein was incubated with DMSO (left panels) or PN-1 (right panels) at 4 °C for 24 h. Green fragment ion peaks correspond to b-ions; orange fragment ion peaks represent y-ions.

**Figure S14**


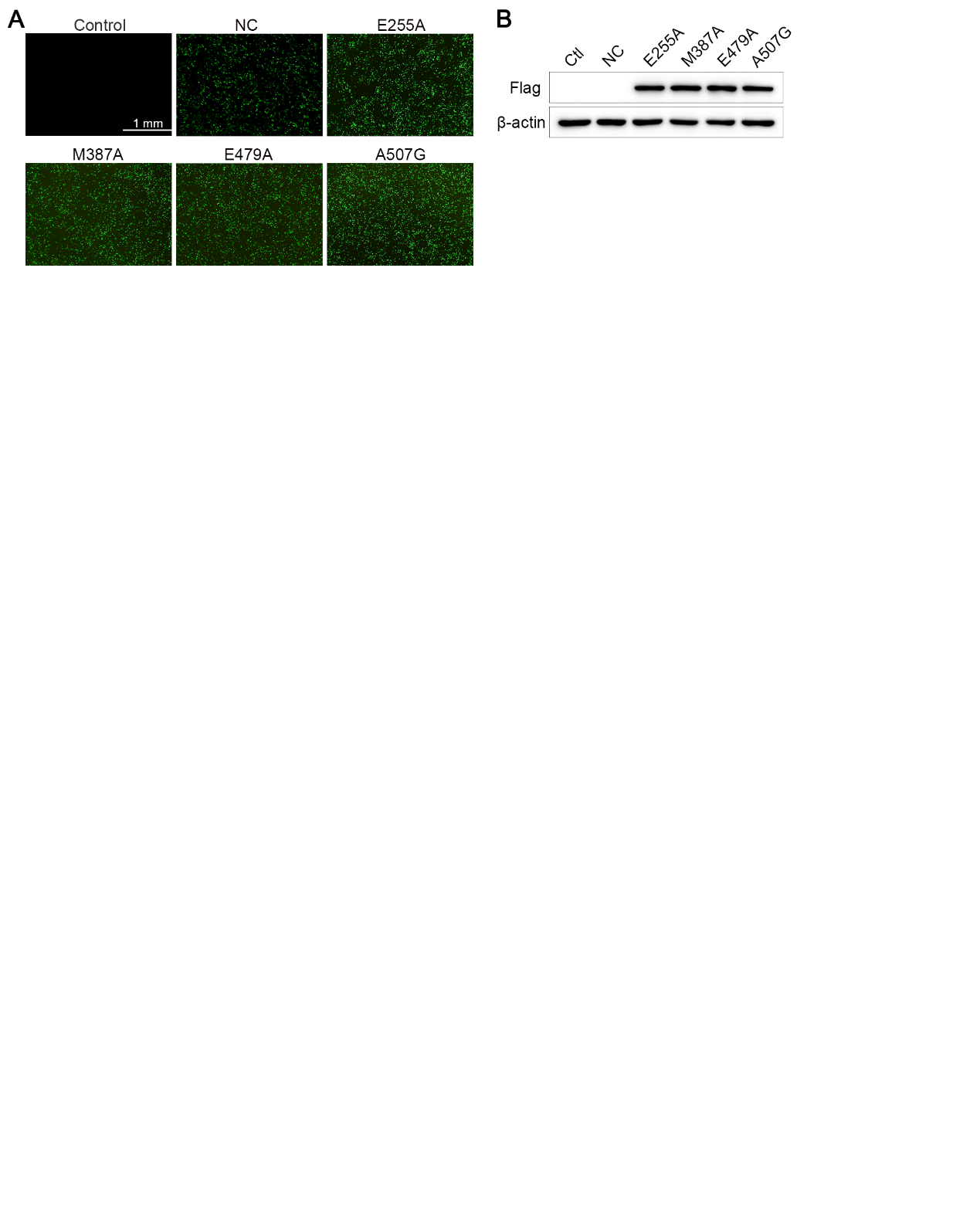


**Figure S14** The transfection efficiency of E255A, M387A, E479A and A507G plasmids was detected by immunofluorescence assay (A) and Western blot analysis (B).

**Figure S15**


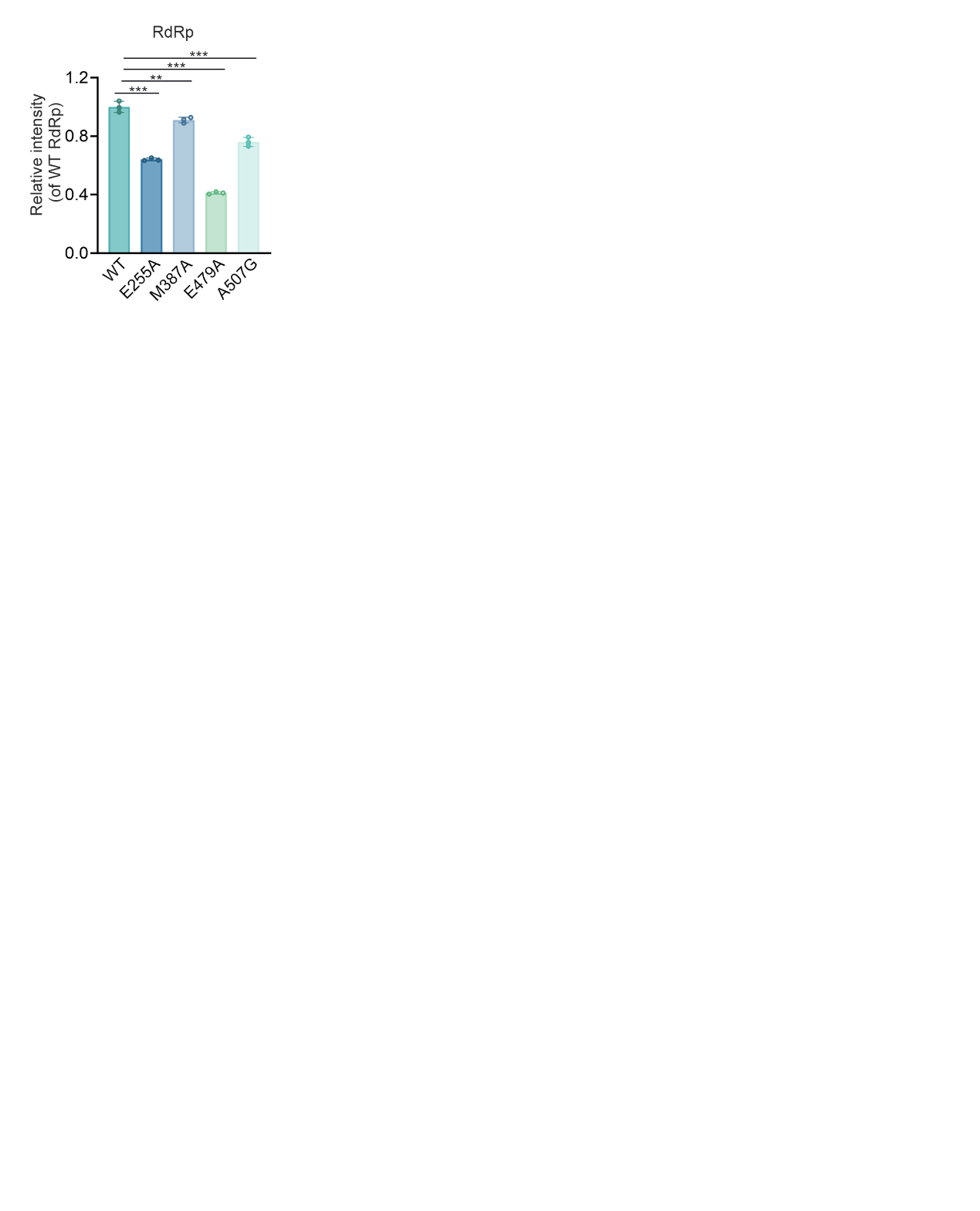


**Figure S15** Quantification of RdRp protein levels from pull down assays using WT or mutated RdRp plasmids transfected cell lysates. Data from Western blot densitometry. ^**^*P* < 0.01, ^***^*P* < 0.001.

**Figure S16**

**
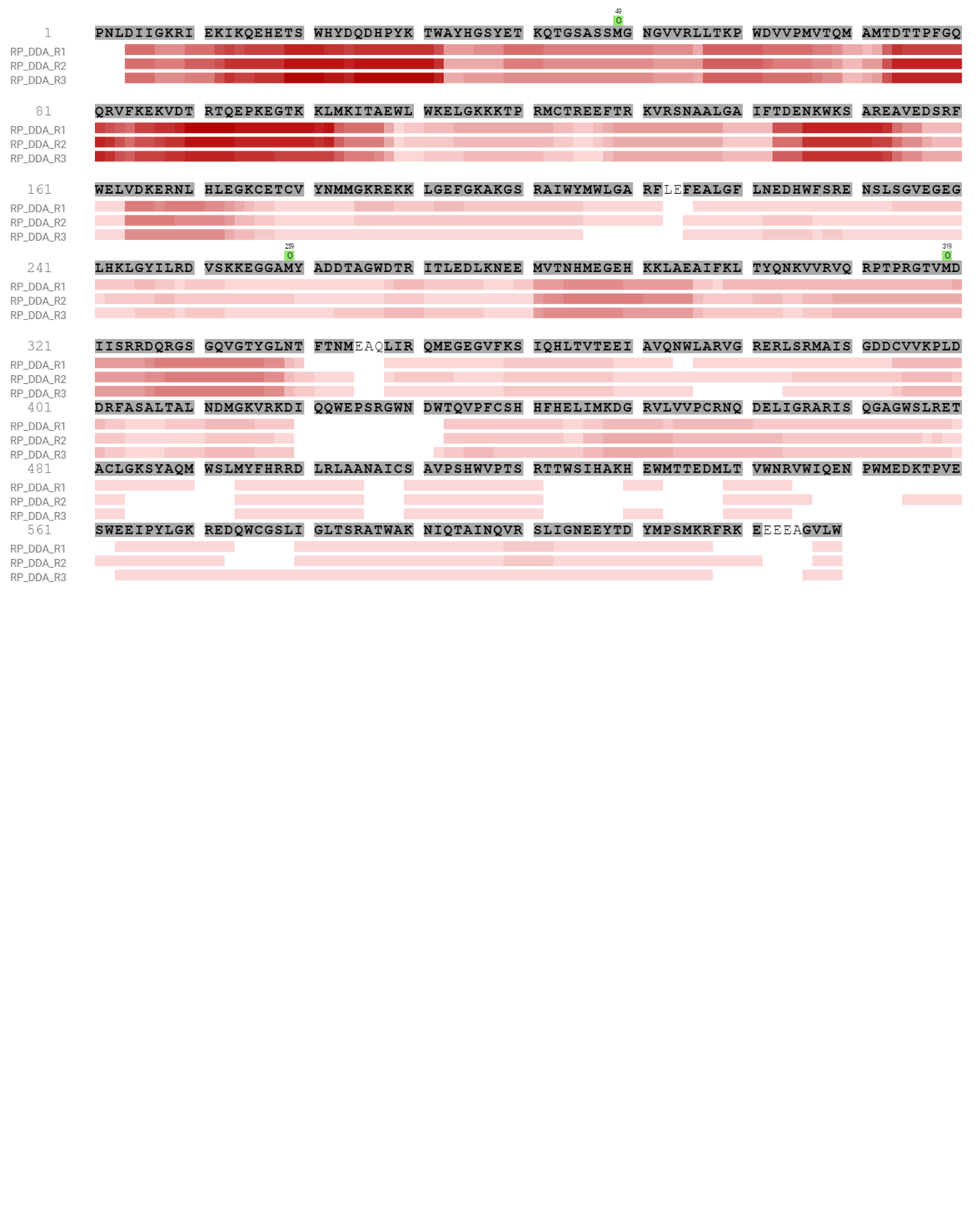
**

**Figure S16** The peptide coverage of RdRp in hydrogen-deuterium exchange-mass spectrometry.

**Figure S17**

**
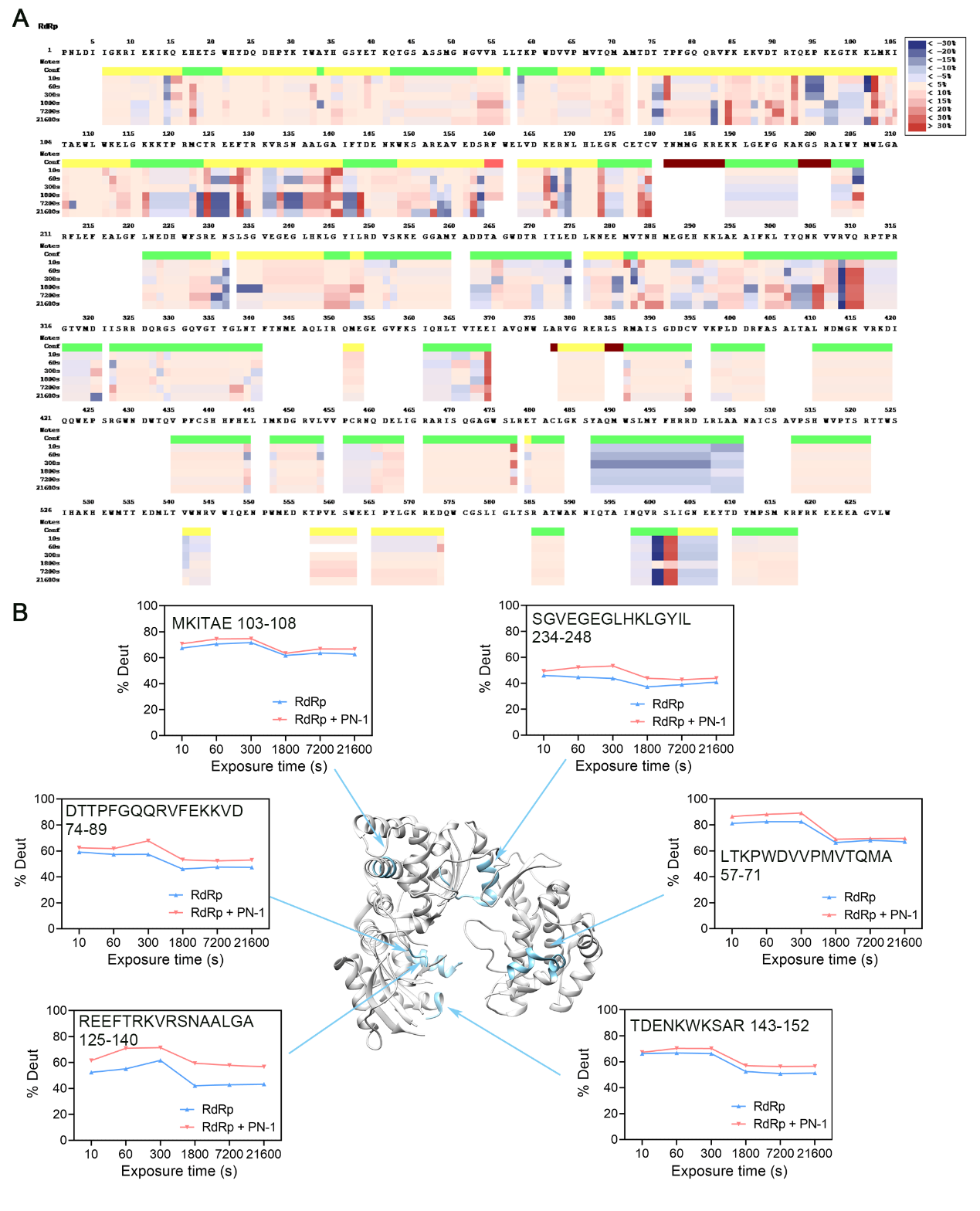
**

**Figure S17** The dynamic changes of RdRp after PN-1 treatment using hydrogen-deuterium exchange-mass spectrometry. (A) Heatmap of PN-1 and RdRp at all hydrogen deuterium exchange time points. (B) The peptides with higher levels of hydrogen–deuterium exchange after PN-1 treatment have been marked in blue.

**Figure S18**


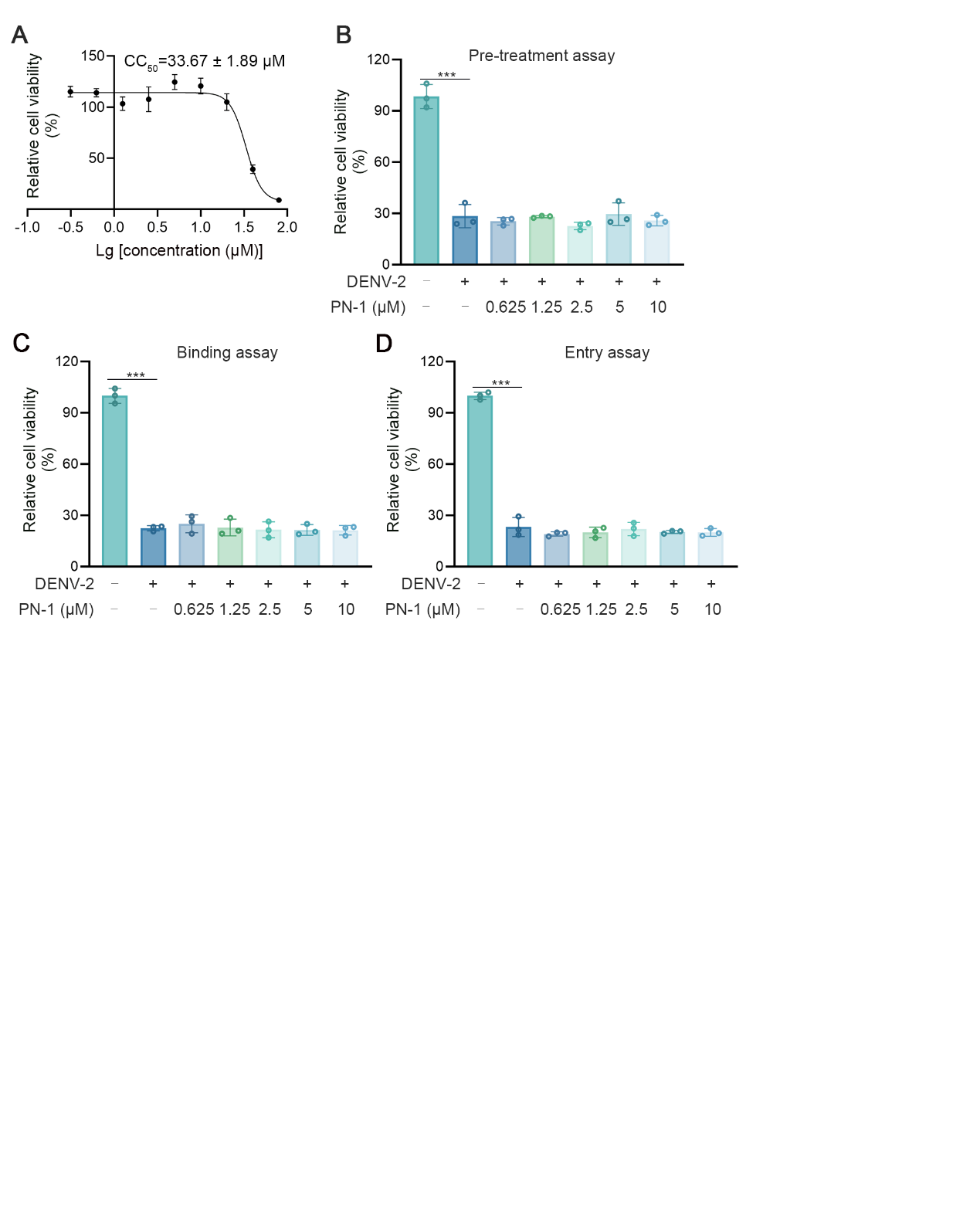


**Figure S18** PN-1 does not inhibit DENV-2 attachment/entry in BHK-21 cells. (A) Relative cell viability of BHK-21 cells under PN-1 treatment. PN-1 had no obvious effect on DENV-induced cell death when administered pre-infection (B), at the viral adsorption phase (C), or at the entry phase (D). The cell viability of BHK-21 cells was detected by CCK-8 assay. ^***^*P* < 0.001.

**Figure S19**


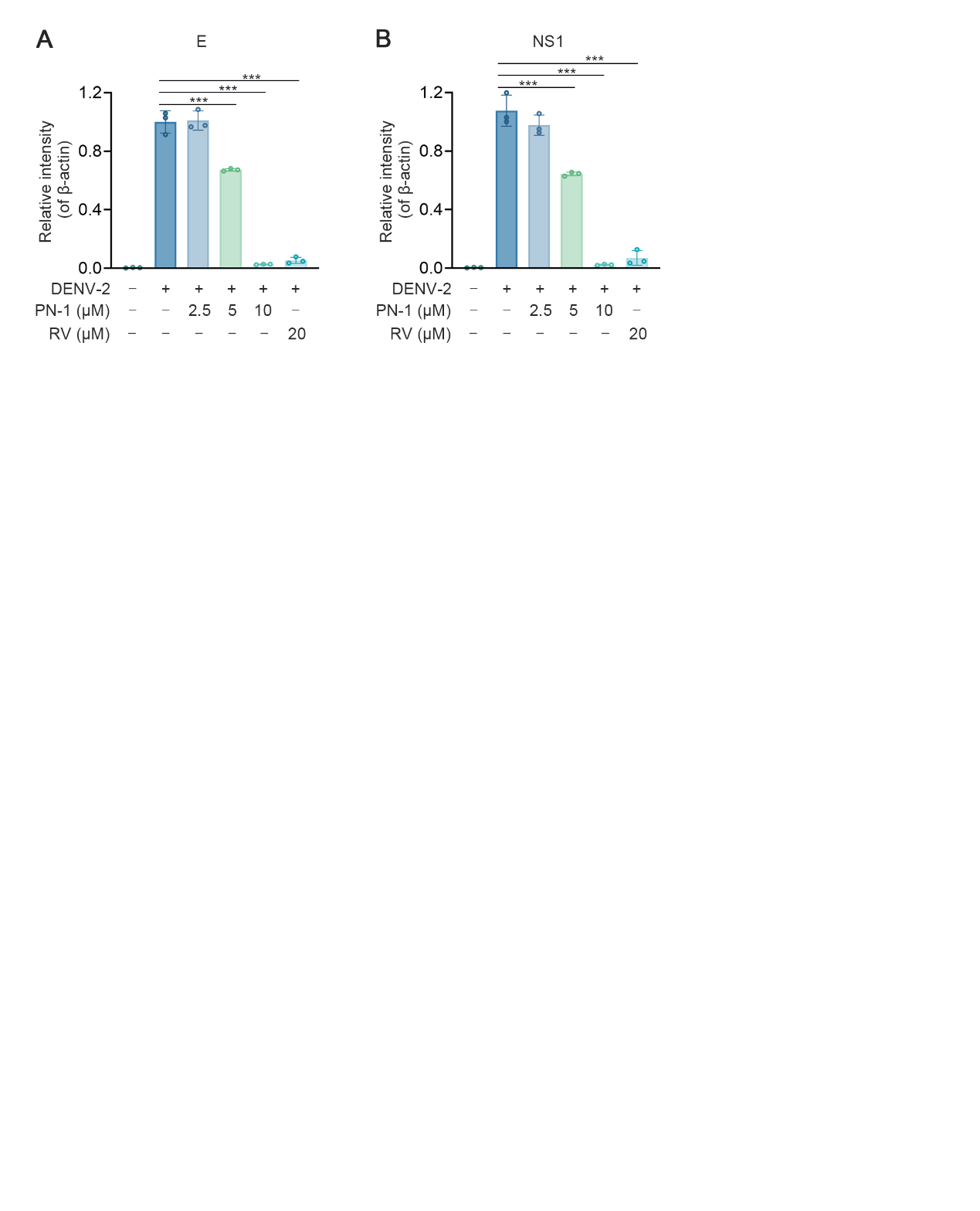


**Figure S19** PN-1 suppresses DENV-2 protein expression in BHK-21 cells. E (A) and NS1 (B) protein levels in cells infected with DENV-2 and treated with PN-1. Quantified by Western blot densitometry. ^***^*P* < 0.001.

**Figure S20**


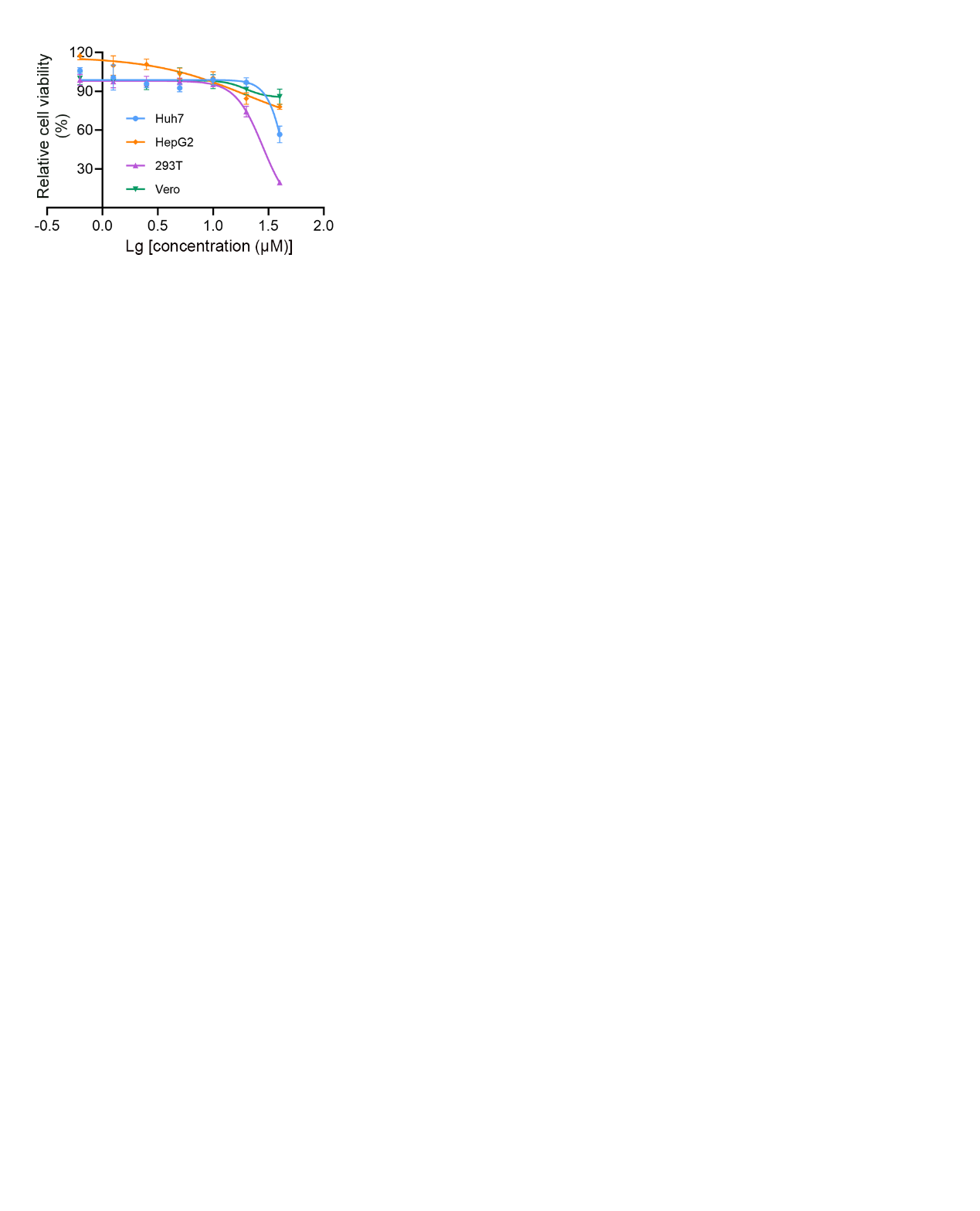


**Figure S20** Effects of PN-1 on the cell viability of Huh7, HepG2, 293T and Vero cells assessed by MTT assay.

**Figure S21**


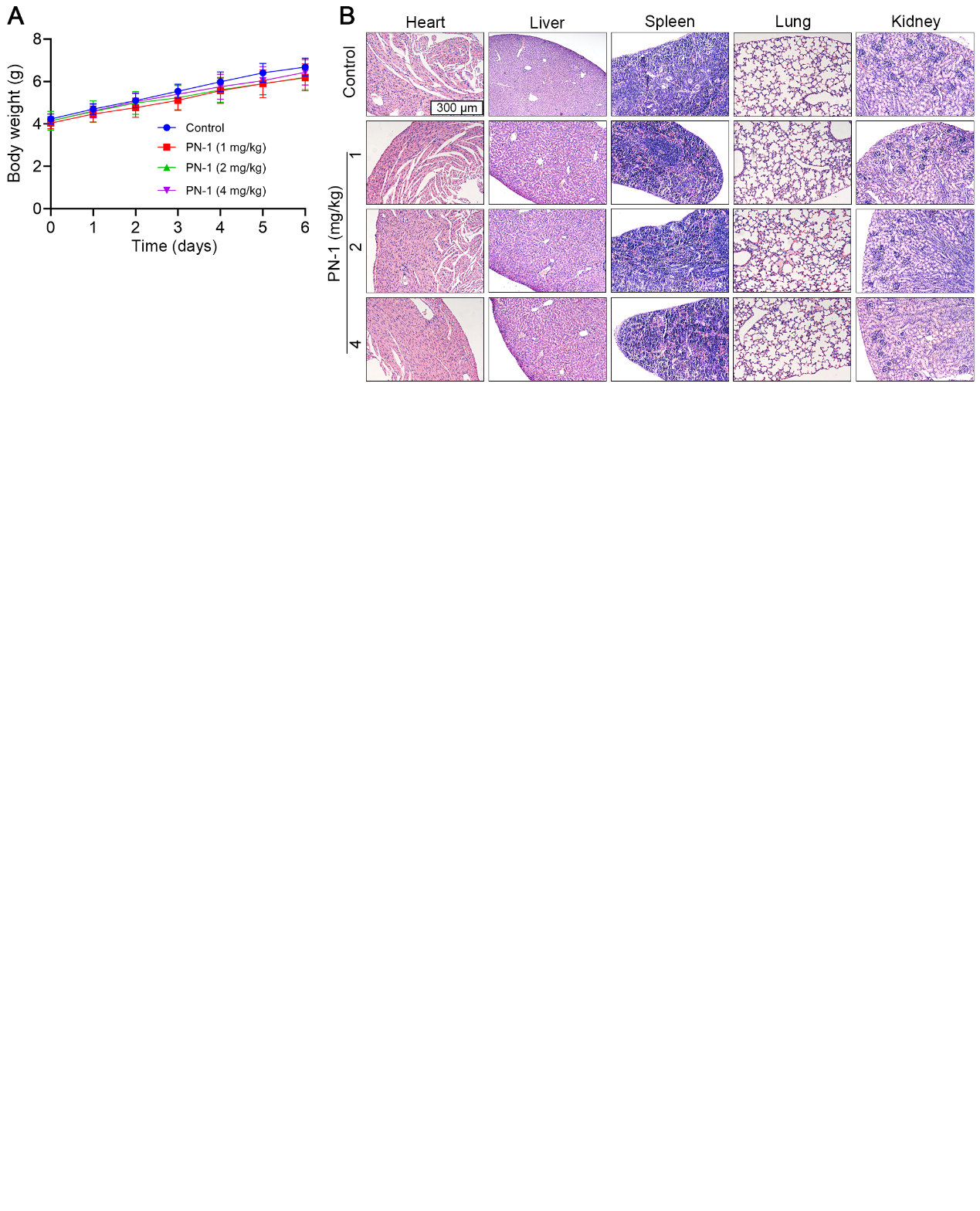


**Figure S21** Safety assessment of PN-1 in ICR suckling mice. (A) Body weight changes during 6-day treatment with PN-1 (1, 2, 4 mg/kg/day, i.p.) or vehicle. (B) Representative H&E-stained sections of heart, liver, spleen, lung and kidney tissues. No significant histopathological alterations observed compared to untreated controls.

**Figure S22**

**
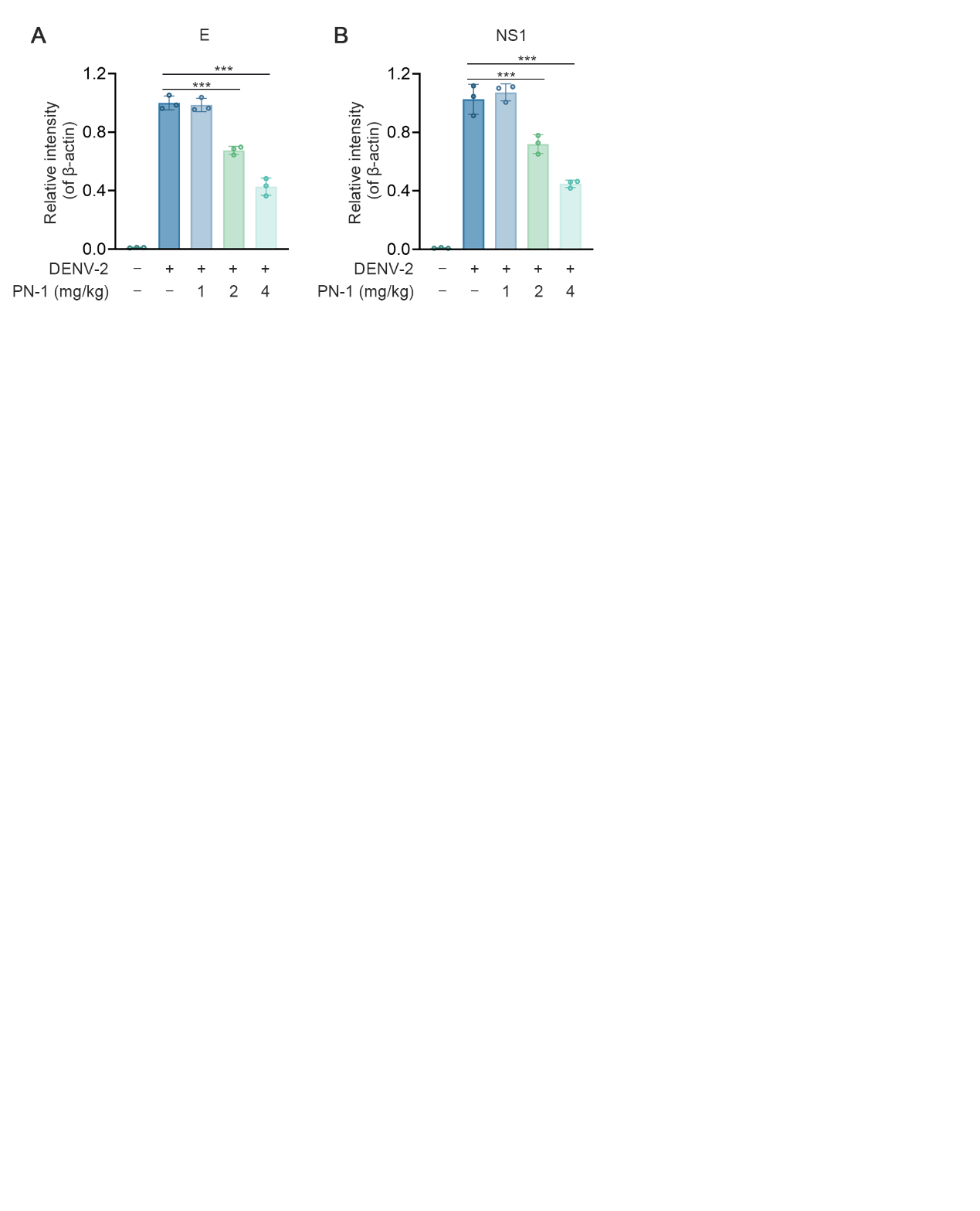
**

**Figure S22** PN-1 suppresses DENV-2 replication in mouse brain. (A-B) Quantification of viral E (A) and NS1 (B) protein levels in brain tissues of ICR suckling mice infected with DENV-2 and treated with PN-1. Data from Western blot densitometry. ^***^*P* < 0.001.

**Figure S23**

**
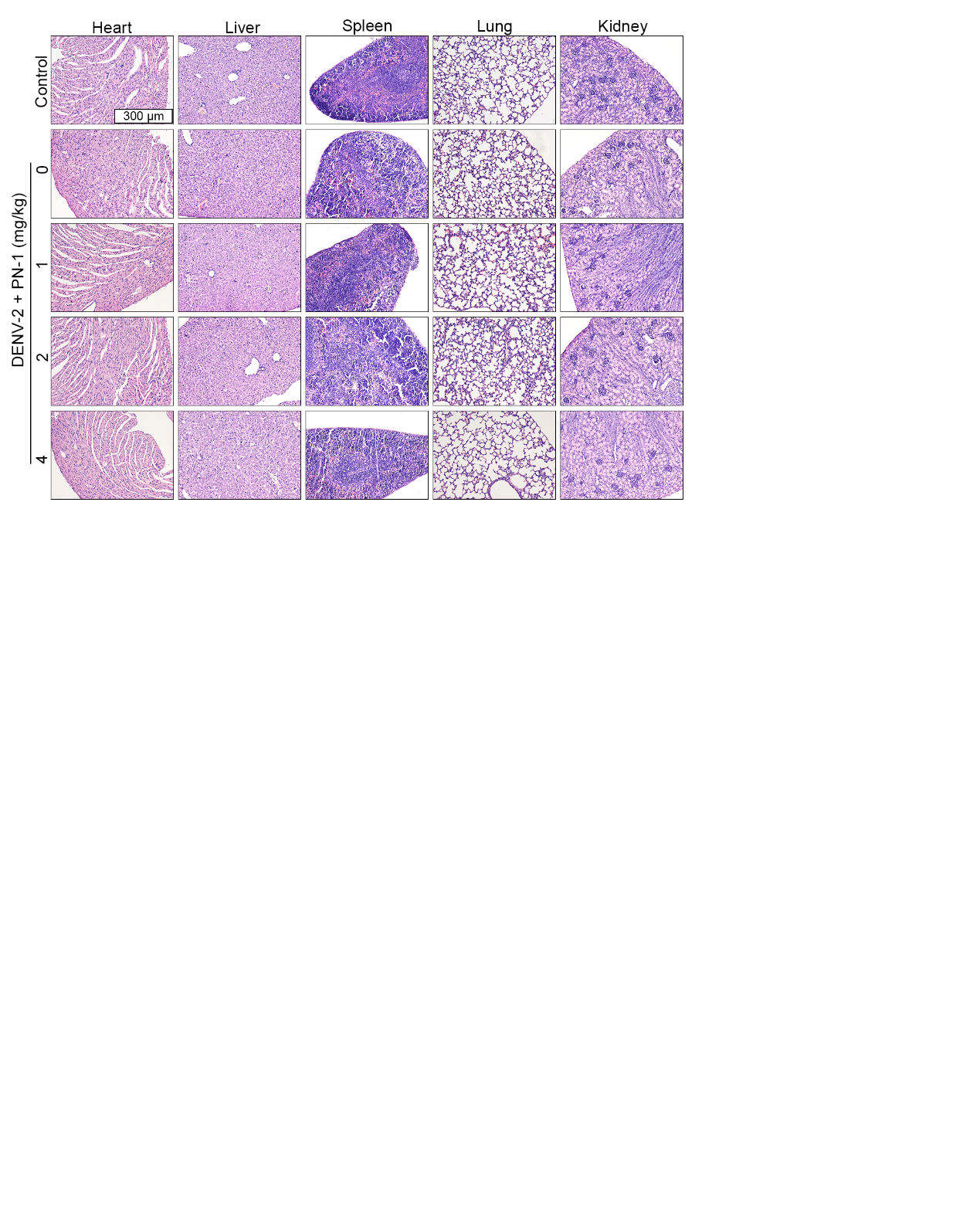
**

**Figure S23** Histopathological analysis of heart, liver, spleen, lung and kidney tissues from DENV-2-infected ICR suckling mice. Tissues harvested under anesthesia show no significant alterations compared to uninfected controls.
